# Incidence and prevalence of autoimmune diseases in China: A systematic review and meta-analysis of epidemiological studies

**DOI:** 10.1016/j.gloepi.2024.100158

**Published:** 2024-08-09

**Authors:** Olaa Mohamed-Ahmed, Lianhan Shang, Lin Wang, Zhengming Chen, Christiana Kartsonaki, Fiona Bragg

**Affiliations:** a Clinical Trial Service Unit and Epidemiological Studies Unit, Nuffield Department of Population Health, University of Oxford, Oxford, OX3 7LF, UK; b UK Health Security Agency, London SW1P 3JR, UK; c Medical Research Council Population Health Research Unit, Nuffield Department of Population Health, University of Oxford, Oxford, OX3 7LF, UK

**Keywords:** Autoimmunity, Autoimmune diseases, Autoimmune conditions, Epidemiology, China, Crohn's disease, Ulcerative colitis, Multiple sclerosis, Graves' disease, Hashimoto disease

## Abstract

**Background:**

Autoimmune diseases account for a substantial burden of disease in high-income countries, including Europe and North America. However, their epidemiology remains under-researched in other regions. We examined the incidence and prevalence of eight autoimmune diseases in the adult Chinese population through a systematic review of epidemiological studies.

**Methods:**

We searched OvidSP MEDLINE and Google Scholar from 1995 to 2023 (inclusive) for articles on the incidence or prevalence of autoimmune thyroiditis (AT), Graves' disease (GD), type 1 diabetes mellitus (T1D), multiple sclerosis (MS), Crohn's disease (CD), ulcerative colitis (UC), rheumatoid arthritis (RA) and systemic lupus erythematosus (SLE). We included studies from mainland China, Taiwan, Hong Kong or Macau. The study is registered with PROSPERO (CRD42021225842).

**Findings:**

We retrieved 2278 records, of which 62 studies (161 estimates) were included in the systematic review, and 42 studies (101 estimates) were included in the meta-analysis. Pooled fixed-effects estimates for incidence of CD, UC, MS, T1D and SLE were 0.22 (95% CI 0.21–0.23), 1.13 (1.10–1.17), 0.28 (0.26–0.30), 2.20 (1.70–2.84) and 4.87 (4.21–5.64) per 100,000 persons, respectively. For RA, one study estimate was included, with an incidence of 15.8 per 100,000 persons. Fixed-effects estimates for the prevalence of CD, UC, MS, SLE, RA, GD and AT were 3.73 (95% CI 3.68–3.78), 16.11 (15.93–16.29), 4.08 (3.95–4.21), 93.44 (92.27–94.63), 104 (103–106), 450 (422–481) and 2322 (2057-2620), respectively, per 100,000 persons. Across all conditions, women were almost twice as likely as men to be diagnosed with an autoimmune disease.

**Interpretation:**

There is marked variation in the frequency of autoimmune diseases among Chinese adults. We estimate that 2.7–3.0% (>31 million people) of the adult Chinese population have one or more autoimmune diseases, comparable to Western populations, with the majority of the burden from autoimmune thyroid diseases and rheumatoid arthritis.

## Introduction

Autoimmune diseases comprise a diverse group of conditions characterised by failure of the body to distinguish self from non-self, leading to damage to specific organs or systemic effects [1]. Although individual diseases are rare, over 100 autoimmune diseases have been characterised [1], which collectively represent a significant disease burden [2]. The onset of most autoimmune diseases is in youth or middle age, and they are typically chronic, associated with multi-morbidity, lifelong disability and high healthcare costs [3,4]. There is a need to understand the burden and pathophysiology of these conditions and effective approaches for preventation.

Historically, autoimmune diseases were considered to primarily affect white people of European descent living in high-income countries (e.g., in Europe, North America and Australia) [5], where 3–9% of the population are estimated to have an autoimmune disease [[6], [7], [8]]. More recently, there has been increasing recognition of a high prevalence of autoimmune diseases in some ethnic groups in these high-income countries [[9], [10], [11]]. However, the diseases remain under-recognised and under-researched in these same ethnic groups in their countries of origin in Africa and Asia.

Rapid economic development, industrialisation, urbanisation and modernisation in China has been associated with increasing adoption of “Western” diets and lifestyles [12], accompanied by an epidemiological transition towards non-communicable diseases [13]. However, it is unclear what proportion of this non-communicable disease burden results from autoimmune diseases. A 2009 comprehensive review of the global literature on autoimmune disease epidemiology identified only a few studies in China, each reporting on an individual condition [6]. Since then, there has been a growing number of published epidemiological studies, particularly on inflammatory bowel diseases, in China and the Asia-Pacific region [14,15], which have shown increasing burden. However, studies on other conditions remain sparse and, indeed, no studies have systematically examined the epidemiology of multiple autoimmune diseases in China. As such, this study aims to establish the incidence and prevalence of eight of the most common, well-characterised autoimmune diseases among adults in China, based on a systematic review of published articles.

## Methods

We conducted a systematic review and meta-analysis of observational studies in accordance with PRISMA and MOOSE guidelines [16,17]. The protocol for this review was published in the PROSPERO database, University of York (CRD42021225842) on 22nd June 2021, prior to commencement of full text screening and data extraction.

### Search strategy and selection criteria

We electronically searched the literature using OvidSP MEDLINE (1995 to 2021) and Google Scholar (1995 to 2021) on 31st March 2021 for articles on autoimmune thyroiditis (AT), Graves' disease (GD), type 1 diabetes (T1D), multiple sclerosis (MS), Crohn's disease (CD), ulcerative colitis (UC), inflammatory bowel disease (IBD), rheumatoid arthritis (RA) or systemic lupus erythematosus (SLE). For the MEDLINE search, we used free text, MeSH and keyword subject headings (**Supplementary Table 1**). For the Google Scholar search, we included the first 50 articles of each of 11 searches conducted in “incognito” mode. Duplicate entries with matching authors, year, title and journal were identified using Endnote 20 (Clarivate, Philadelphia, PA) and Rayyan software, and were excluded after review. Two authors (OMA, FB) independently screened each title and abstract; any differences were resolved through discussion and review of the full text. The OvidSP MEDLINE search was updated on 9th February 2024, to include any articles published from 2021 to 2023, inclusive.

Studies were included in the systematic review if they met all of the following criteria: original article published in a peer-reviewed journal; observational study reporting empirical data on the incidence or prevalence of one of more autoimmune diseases of interest or data enabling calculation of these metrics; reporting on disease burden in China (including mainland China, Hong Kong, Macau and Taiwan) (global or regional studies were included if they presented data separately for China); English or Chinese language. For inclusion in the meta-analysis, articles needed to present an annual incidence or period prevalence estimate, in persons (rather than person-years), or provide sufficient data for this to be calculated.

Studies were excluded if they: focused solely on a paediatric population (≤18) or on a non-Chinese or immigrant population in mainland China/Hong Kong/Macau/Taiwan, or a Chinese population outside of these locations; selected participants on the basis of pre-existing disease (with the exception of the specified diseases of interest); or were animal studies, systematic reviews, meta-analyses, intervention studies or case series. No study investigators were contacted for further details. Reference lists of all included articles were searched for additional relevant studies. Abstracts of snowballed articles were reviewed and included in the systematic review if they met the inclusion criteria.

Where studies used duplicate data (exactly the same study location, cases and population), the study presenting the most information (e.g., incidence, prevalence, mean age, gender ratio) was selected for inclusion. For the meta-analysis, where studies used partially overlapping data from the same population or data source, the study with the greatest population size was selected. For studies with the same (or similar) population size or using the same database, the study with the most recent time period was selected.

### Data analysis

Data from each included article were independently extracted by two authors (English articles: OMA and FB or CK, Chinese articles: LS and LW) using a data extraction form which included study characteristics (publication date, data source, study location, cases and population, autoimmune disease studied) and epidemiological findings (incidence, prevalence, year(s) covered, mean age, and gender ratio). Each author assessed the risk of bias and quality of the evidence independently using the Joanna Briggs Institute (JBI) Critical Appraisal Checklist for studies reporting prevalence and incidence data [18]; each of the nine questions was scored as yes, no or unsure. Areas of disagreement or uncertainty were discussed to reach a consensus.

We defined an estimate as a measure in a defined population; where studies presented this by a geographic location, year or subgroup (e.g. by ethnicity, age or exposure of interest), we included each as a separate point estimate. Crude or standardised incidence and prevalence estimates were included. For studies over multiple years, the average annual incidence was chosen. If this was not available, in order of preference, the most recent annual incidence or cumulative incidence was selected. Where studies did not present 95% confidence intervals, they were calculated using the standard error (SE) of the incidence, approximated using the average population rather than person-years, [SE = (√cases)/population] and/or the prevalence [SE = √ ((prevalence × (1-prevalence))/population)]. Where studies did not present a gender ratio, this was calculated (in order of preference) as a rate ratio or case ratio depending on the availability of data. For the meta-analysis, we excluded any estimates presented in person-years, because the majority of estimates were available in “persons” only.

The data source was classified as active surveillance or registry (if there was any active case finding), routine healthcare data, or survey (if a community-based population sample was selected). We accepted a range of diagnostic classifications for the autoimmune conditions including ICD-9, ICD-10, international criteria (e.g. Lennard-Jones, American College of Rheumatology), clinical diagnosis, and self-report. We did not exclude any studies based on quality assessment.

Meta-analysis was conducted using R version 4.1.1, RStudio version 1.4.1717, packages ‘meta’ [19], ‘ckbplotr’ [20] and ‘ggplot2’ [21]., We used fixed-effects inverse-variance weighted average and random-effects methods with logarithm of the incidence and logit of prevalence to give pooled estimates for each autoimmune disease. Where only one estimate was identified, no meta-analysis was conducted and if only two studies were identified, no random-effects estimate was calculated. Where five or fewer estimates were identified, a Hartung-Knapp adjustment was used to generate the random-effects pooled estimate [22,23]. We assessed statistical heterogeneity using I^2^, and Cochran's Q [24]. Funnel plots were used to assess for publication bias, with the log odds plotted against the study size for all estimates, and by autoimmune condition [25].

### Role of the funding source

The funder had no role in study design, data collection, data analysis, data interpretation, or writing of the report.

## Results

**Fig. 1:**
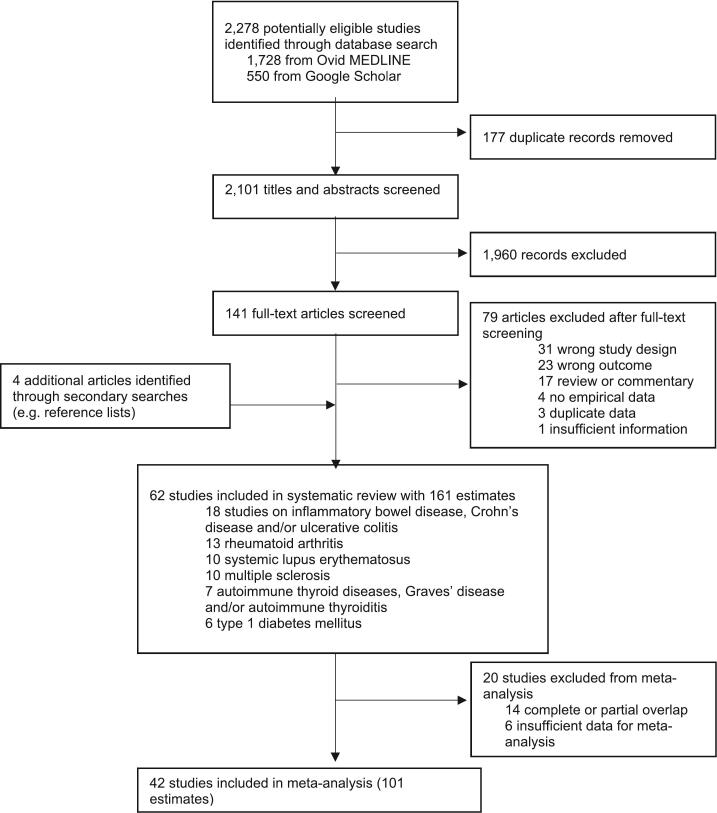
Study selection. Full-text articles could have been excluded for more than one reason; only the primary reason for exclusion is listed here. Some studies included more than one autoimmune condition, and, thus, the sum of the number of studies included for each autoimmune condition does not equal the total number of included studies. *Adapted From:* Page MJ, McKenzie JE, Bossuyt PM, Boutron I, Hoffmann TC, Mulrow CD, et al. The PRISMA 2020 statement: an updated guideline for reporting systematic reviews. BMJ 2021;372:n71 [16].

Overall, 2278 records were retrieved, of which 62 studies and 161 estimates of incidence/prevalence met the inclusion criteria (Fig. 1). Of these, 42 (67.7%) studies and 101 (62.7%) estimates were included in the meta-analysis, with the main reasons for exclusion being complete or partial overlap of the population and/or data source used for the study (*n* = 14) or insufficient data (*n* = 6). Full details, characteristics and quality assessment of each included study are available in **Supplementary Tables 2–4**.

The 62 studies identified included 566,268 cases of autoimmune disease (Table 1). The greatest number of cases were identified for UC (*n* = 151,328), and the lowest for AT (*n* = 326) and GD (*n* = 914), which were studied in the smallest populations (31,000 and 225,000, respectively). Most commonly, the data sources from which estimates were generated involved routine healthcare data (*n* = 62, 39%) and active surveillance or disease registries (*n* = 61, 38%), followed by surveys (*n* = 38, 24%). Surveys were most frequently used to estimate the burden of AT, GD and RA (100%, 100% and 58% of estimates, respectively).

**Table 1:** Characteristics of estimates included in the systematic review (*n* = 161).

Characteristic	Crohn's disease, *N* = 30	Ulcerative colitis, *N* = 33	Inflammatory bowel disease, *N* = 18	Multiple sclerosis, *N* = 12	Type 1 diabetes, N = 8	Systemic lupus erythematosus, *N* = 16	Rheumatoid arthritis, *N* = 19	Graves' disease, *N* = 14	Autoimmune thyroiditis, *N* = 11	TOTAL, N = 161
*DEMOGRAPHICS*										
Cases†	25,628	151,328	104,325	19,891	52,825	81,301	129,730	914	326	566,268
Population†, in 000 s	1,572,642	1,580,081	724,300	1,570,996	280,201	116,886	94,285	225	31	5,939,647
Age‡	33.0	43.0	40.0	40.4	32.5	35.7	51.1	44.2	Not stated	41.0
Gender ratio, Female-to-Male ‡	0.5	0.7	0.7	3.2	1.0	7.2	3.2	2.4	2.4	1.8
STUDY LOCATION*										
Mainland China	15 (50%)	15 (45%)	11 (61%)	4 (33%)	2 (25%)	3 (19%)	11 (58%)	14 (100%)	11 (100%)	86 (53%)
Hong Kong	6 (20%)	8 (24%)	3 (17%)	2 (17%)	1 (13%)	2 (13%)	0 (0%)	0 (0%)	0 (0%)	22 (14%)
Macau	1 (3·3%)	1 (3·0%)	1 (5·6%)	0 (0%)	0 (0%)	0 (0%)	0 (0%)	0 (0%)	0 (0%)	3 (1.9%)
Taiwan	7 (23%)	8 (24%)	2 (11%)	6 (50%)	5 (63%)	11 (69%)	8 (42%)	0 (0%)	0 (0%)	47 (29%)
Combined §	1 (3·3%)	1 (3·0%)	1 (5·6%)	0 (0%)	0 (0%)	0 (0%)	0 (0%)	0 (0%)	0 (0%)	3 (1.9%)
DATA SOURCE*										
Active surveillance or registry (AS/R)	16 (53%)	17 (52%)	16 (89%)	6 (50%)	3 (38%)	3 (19%)	0 (0%)	0 (0%)	0 (0%)	61 (38%)
Routine healthcare data (RH)	14 (47%)	16 (48%)	2 (11%)	6 (50%)	5 (63%)	11 (69%)	8 (42%)	0 (0%)	0 (0%)	62 (39%)
Survey (S)	0 (0%)	0 (0%)	0 (0%)	0 (0%)	0 (0%)	2 (13%)	11 (58%)	14 (100%)	11 (100%)	38 (24%)
*STUDY YEARS OF DATA COLLECTION* *¶										
1980–1989	2 (6·7%)	6 (18%)	2 (11%)	1 (8·3%)	0 (0%)	0 (0%)	1 (5·3%)	0 (0%)	0 (0%)	12 (5.4%)
1990–1999	10 (33%)	14 (42%)	2 (11%)	3 (25%)	1 (13%)	0 (0%)	2 (11%)	7 (50%)	6 (55%)	45 (20%)
2000–2009	12 (40%)	16 (48%)	3 (17%)	6 (50%)	7 (88%)	13 (81%)	10 (53%)	3 (21%)	3 (27%)	74 (33%)
2010–2019	23 (77%)	24 (73%)	17 (94%)	5 (42%)	6 (75%)	4 (25%)	2 (11%)	4 (29%)	2 (18%)	89 (40%)
2020–2023	0 (0%)	0 (0%)	0 (0%)	1 (8.3%)	0 (0%)	1 (0.6%)	0 (0%)	0 (0%)	0 (0%)	2 (0.9%)

*n (%); †Sum; ‡Median. Note for age this has been determined based on the mean or median age presented for the estimate. The number of estimates with missing data was 78 and 63 for Age and Gender ratio, respectively; § estimates including combined data from all four locations (Mainland China, Hong Kong, Taiwan and Macau); ¶ each estimate could be included in more than one category for this variable if the study spanned more than one decade; Gender ratio includes case, incidence and prevalence ratios.

The majority of estimates were from mainland China (*n* = 86, 53%), followed by Taiwan (*n* = 47, 29%) and Hong Kong (*n* = 22, 14%), with only three estimates from Macau (1.9%). The majority of estimates were from recent decades, 2010–2019 (*n* = 89, 40%) and 2000–2009 (*n* = 74, 33%). The mean age across all conditions was 39.8, though for almost half of the estimates (*n* = 78, 48.4%), the average age was not presented. A gender ratio was presented or could be calculated for 98 estimates (60.9%), showing the burden was almost twice as high in women than in men across all conditions when combined. The gender ratio was highest for SLE, which was seven times higher in women compared to men; while for CD, UC, IBD, the burden was higher in men; the burden of T1D was equal between men and women (Table 1).

**Fig. 2:**
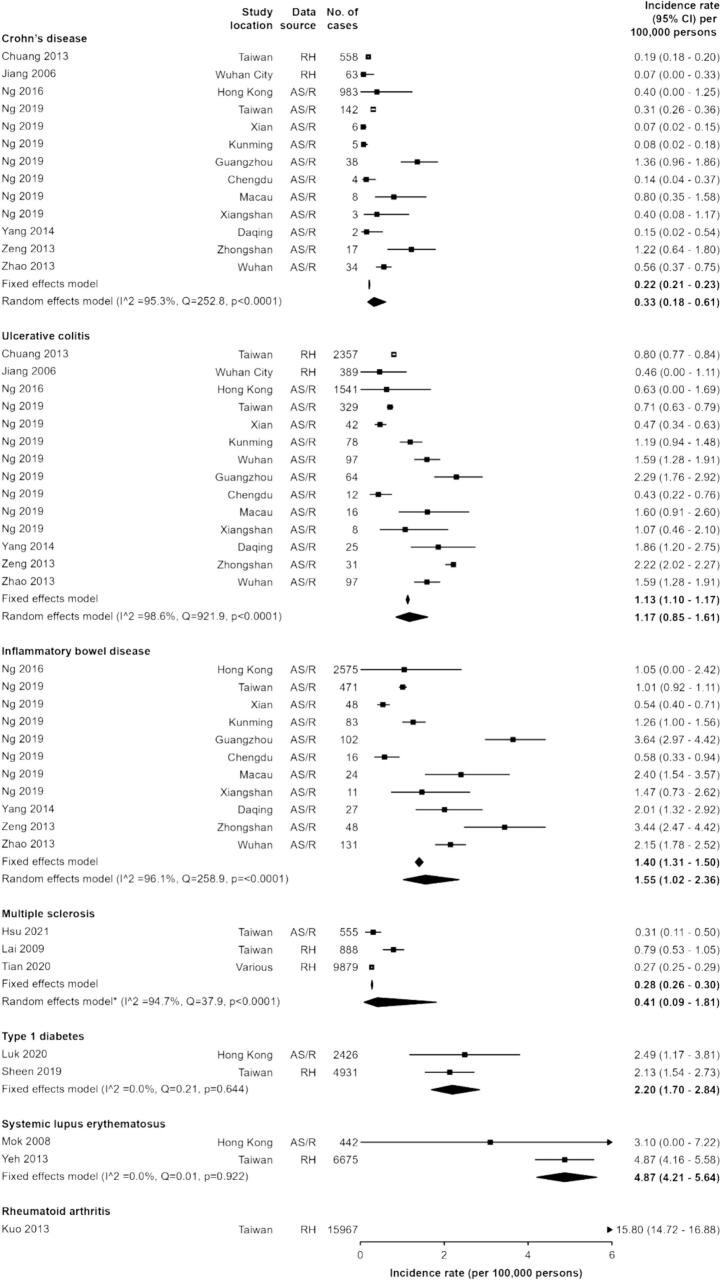
Incidence of autoimmune diseases. * Standardised incidences presented for Ng 2016 and Tian 2020. All other studies present crude annual incidence. AS/R = Active surveillance or registry, RH = Routine healthcare data, S = Survey.

For CD, UC, IBD, MS, T1D and SLE, all estimates identified were below 5 per 100,000 persons (Fig. 2), and all were based on studies using active surveillance or registry (AS/R) or routine healthcare (RH) data. For CD (13 estimates; 7 studies), the pooled incidence estimate was 0.22 (95% CI 0.21–0.23) per 100,000 persons in the fixed-effects model and 0.33 (0.18–0.61) per 100,000 persons in the random-effects model [[26], [27], [28], [29], [30], [31]]. For UC (14 estimates; 7 studies), the pooled fixed-effects and random-effects estimates were 1.13 (1.10–1.17) and 1.17 (0.85–1.61) per 100,000, respectively,[[26], [27], [28], [29], [30], [31]] whilst for IBD as a group (CD, UC +/− IBD-undetermined; 11 estimates; 5 studies), the fixed-effects and random-effects pooled estimates were 1.40 (1.31–1.50) and 1.55 (1.02–2.36) per 100,000 persons, respectively [[27], [28], [29], [30], [31]].

For MS, three estimates were included in the meta-analysis, with the largest study based on 1.2 billion adults in mainland China, with an incidence of 0.27 (0.25–0.29) per 100,000 persons [32]. The remaining studies were from Taiwan, based on 23 million participants [33,34]. These were combined to give [32] a pooled fixed-effects estimate of 0.28 (0.26–0.30) per 100,000 persons and random-effects estimate of 0.41 (0.09–1.81) per 100,000 persons. For T1D and SLE the estimated fixed-effects incidence was 2.20 (1.70–2.84) and 4.87 (4.21–5.64) per 100,000 persons, respectively, based on two studies for each. One study reporting the incidence of RA was identified, which estimated the incidence to be 15.80 (14.72–16.88) per 100,000 persons [35]. For T1D, SLE and RA, 13 other incidence estimates were identified, but 10 were excluded from the meta-analysis due to overlapping data since they were based on the same health insurance database from Taiwan, in similar time periods (**Supplementary Table 2**). For GD and AT, one study by Teng et al. (2006) [36] identified 5-year incidence estimates for both conditions across three different areas in mainland China, giving an estimated annual incidence ranging from 110.2 to 158.4 per 100,000 for GD and 45.3–254.6 per 100,000 for AT (**Supplementary Table 2**). However, this was not included in the meta-analysis due to the extremely small number of cases over five years and insufficient information to calculate the pooled incidence accurately.

**Fig. 3:**
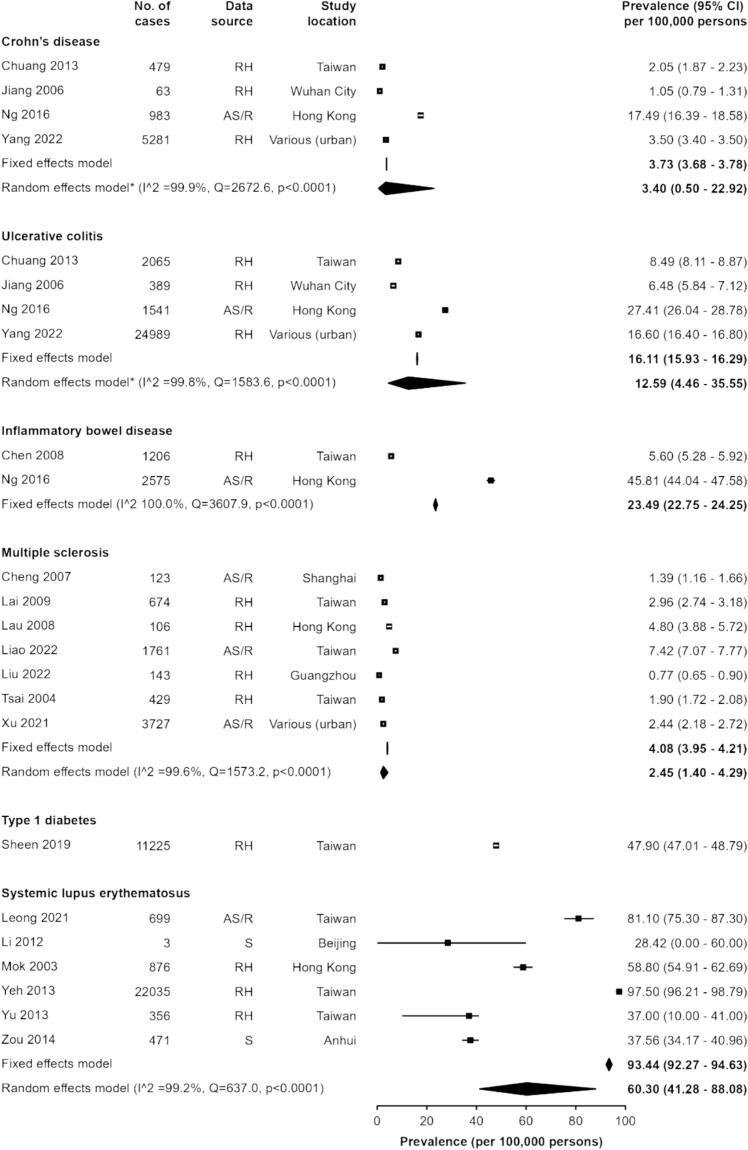
Prevalence of autoimmune diseases (low prevalence). AS/R = Active surveillance or registry, RH = Routine healthcare data, S = Survey.

Due to the difference in the magnitude of estimates between conditions, we grouped conditions into “low prevalence” (CD, UC, IBD, MS, T1D and SLE) and “high prevalence” (RA, GD and AT) groups, based on whether the pooled estimates were lower than 100 per 100,000 persons, or greater, respectively. For CD and UC, the fixed-effects pooled estimates were 3.73 (95% CI 3.68–3.78), 16.11 (15.93–16.29) and random-effects estimates were 3.40 (0.50–22.92) and 12.59 (4.46–35.55) per 100,000 persons, respectively, based on four studies covering Taiwan, Hong Kong and mainland China (Fig. 3) [[26], [27], [37], [38]]. Several other studies were identified but were excluded due to partial overlap or insufficient data (**Supplementary Table 3**). The Hong Kong studies, [27] which used active case finding had higher prevalence than the Taiwan[[26], [39]] and mainland China studies, [[37], [38]] which did not. For MS (7 estimates; 7 studies) the pooled estimates were 4.08 (3.95–4.21) and 2.45 (1.40–4.29) per 100,000 persons in the fixed-effects and random-effects models, respectively [[33], [40], [41], [42], [43], [44], [45]]. One prevalence estimate was identified for T1D of 47.90 (95% CI 47.01–48.79) per 100,000 persons in Taiwan [46]. For SLE (6 estimates; 6 studies) the pooled estimates were 93.44 (92.27–94.63) and 60.30 (41.28–88.08) per 100,000 persons in the fixed-effects and random-effects models, respectively [[47], [48], [49], [50], [51], [52]]. Except for SLE, where two of the studies were based on survey data, all other estimates in the “low prevalence” group were based on active surveillance, registry or routine healthcare data.

**Fig. 4:**
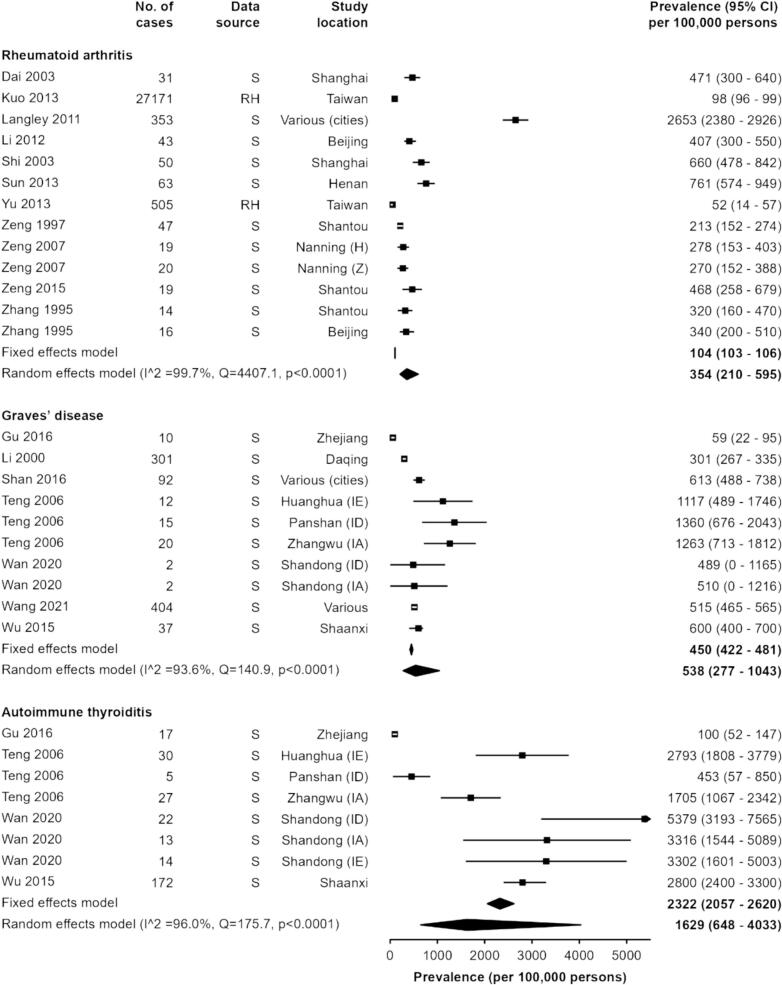
Prevalence of autoimmune diseases (high prevalence). AS/R = Active surveillance or registry, RH = Routine healthcare data, S = Survey, (ID) = iodine deficient, (IA) = iodine adequate, (IE) = iodine excessive, (Z) = Zhuang ethnicity, (H) = Han ethnicity.

In the “high prevalence” group, we identified 13 prevalence estimates (11 studies) for RA, with estimates ranging from 52 to 2653 per 100,000 persons [[35], [47], [50], [53], [54], [55], [56], [57], [58], [59], [60]] (Fig. 4). The majority of estimates were based on survey data, with the notable exception of the two largest studies, which utilised the large routine healthcare datasets from Taiwan in 2002–2007 [35] and 2000 [47], respectively. The pooled estimates of RA prevalence were 104 (95% CI 103–106) and 354 (210–595) per 100,000 persons in the fixed-effects and random-effects models, respectively. Of note, the point estimate from Langley et al. (2011) [53] was a clear outlier.

All studies reporting on autoimmune thyroid disease utilised survey data to generate estimates (Fig. 4). For GD, the estimates ranged from 59 to 1360 per 100,000 persons with pooled estimates of 450 (95% CI 422–481) and 538 (277–1043) per 100,000 persons in the fixed-effects and random-effects models [[36], [61], [62], [63], [64], [65], [66]]. For AT, the estimates were generally much higher than for GD and ranged from 100 to 5379 per 100,000 persons [[36], [61], [62], [66]], with a fixed-effect estimate of 2322 (2057-2620) per 100,000 persons and a random-effects estimate of 1629 (648–4033) per 100,000 persons. Two studies presented the prevalence estimates by iodine intake levels (deficient, adequate and excessive). For both Teng et al. (2006) [36] and Wan et al. (2020) [62] prevalence estimates for GD were similar across all iodine groups. Conversely, for AT, the two studies showed different relationships between prevalence and iodine intake. In Teng et al. (2006) [36] prevalence was highest in the iodine excessive group (Huanghua) at 2793 per 100,000 persons and lowest in the deficient group (Panshan) at 453 per 100,000 persons, whilst in Wan (2020) [62] the prevalence was highest in the iodine deficient group (5379 per 100,000 persons) and lowest in the iodine excessive group (3302 per 100,000 persons). Both studies were very small, and the number of cases identified ranged from 2 to 30 for each estimate.

The I^2^ statistic was over 90% for the majority of autoimmune diseases, suggesting considerable heterogeneity between the estimates for each condition. In general, the studies met most of the JBI quality assessment tool criteria, with the notable exception of many RA studies, which were of lower quality due to sparse information and less robust methods used to sample the population and identify cases (**Supplementary Table 4**). Funnel plots for each of the conditions were generally symmetrical, with the majority of estimates being from studies of a similar size (**Supplementary Fig. 1**).

## Discussion

In this systematic review and meta-analysis, we identified 62 studies and 161 estimates of the incidence or prevalence of eight autoimmune conditions among adults in mainland China, Taiwan, Hong Kong and Macau, published since 1995. We identified studies covering over 566,000 cases, with substantial variation in pooled incidence and prevalence estimates between the eight conditions, ranging from 0.22 (CD) to 15.8 (RA) and 3.73 (CD) to 2322 (AT) per 100,000 persons, respectively. Our findings suggest the incidence and prevalence of many of the autoimmune diseases studied are extremely low or low in China. We found a clear and consistent order in the incidence and/or prevalence of the conditions, with CD the least common, followed by MS, UC, T1D, SLE, RA, GD and AT. Our study shows that autoimmune diseases in China, as elsewhere, predominantly affect women, additionally suggesting there may be regional variations in autoimmune disease burden.

We compared our findings with estimates from North America and Europe (Table 2), focusing on prevalence estimates given their value for understanding burden and impact of chronic diseases. For the least common conditions—CD, UC and MS—the prevalence in China was several orders of magnitude lower than most estimates from North America and Europe, particularly northern and western Europe [[67], [68], [69], [70]]. The studies we identified examining the burden of these three conditions in China were generally of high quality and were based on active surveillance, registries or routine healthcare data from large populations (750,000 to 1.2 billion). Nonetheless, many spanned several decades and it is possible this obscures recent transitions to higher rates. This is supported by studies showing rising incidence of IBD and MS in newly-industrialised nations in Asia and South America, and stable or decreasing rates in North America and Europe, albeit at a far higher level than in the latter [71,72]. However, such temporal trends are unlikely to fully explain the differences between China and Western populations. For T1D and SLE, our pooled prevalence estimates are lower than the highest estimates in North America and Europe [[11], [73]]. For SLE, the range of estimates in China appear to overlap with those in North America and Europe [11]. For T1D, however, the prevalence is more clearly lower than in other regions, whilst SLE prevalence is comparable [11,73].

**Table 2:** Comparison of lowest and highest estimates of autoimmune disease prevalence (per 100,000 persons) in China (this review), North America and Europe.

Autoimmune disease	Prevalence, per 100,000 persons (country)								
	China*			North America†			Europe†		
	Lowest estimate	Highest estimate	Fixed-effects estimate	Lowest estimate	Highest estimate	References	Lowest estimate	Highest estimate	References
Crohn's disease	1.05 (Taiwan)	17.49 (Hong Kong)	3.73	96.3 (USA)	319 (Canada)	[67]	1.51 (Romania)	322 (Germany)	[67]
Ulcerative colitis	6.19 (Taiwan)	27.4 (Hong Kong)	16.1	139.8 (Canada)	286 (USA)	[67]	2.42 (Romania)	505 (Norway)	[67]
Multiple sclerosis	0.77 (Guangzhou)	7.42 (Taiwan)	4.08	42.8 (USA)	364 (Canada)	[70]	15.0 (Spain)	253 (Sweden)	[69]
Type 1 diabetes	26.9 (Taiwan)	47.9 (Taiwan)	47.9	57 (USA) ‡	570 (USA) ‡	[73]	37 (Lithuania) ‡	22,000 (Scotland) ‡	[73]
Systemic lupus erythematosus	28.4 (Beijing)	97.5 (Taiwan)	93.4	48 (Canada)	301 (USA)	[11]	29.3 (Malta)	210 (Spain)	[11]
Rheumatoid arthritis	52 (Taiwan)	2653 (Mainland China)	104	400 (Mexico)	1070 (USA)	[74]	310 (France)	1900 (Finland)	[75]
Graves' disease	59 (Mainland China)	1359 (Mainland China)	450	100 (USA) §	500 (USA) §	[8]	200 (Spain)	2900 (Italy)	[8,76]
Autoimmune thyroiditis	100 (Mainland China)	5379 (Mainland China)	2322	400 (USA)	22,400 (USA)	[77]	400 (Bosnia & Herzegovina)	35,500 (Italy)	[77]

*Lowest and highest estimates are based on all prevalence estimates identified through the systematic review (see Supplementary Table 3). Fixed-effects pooled prevalence estimates are based on estimates included in the meta-analysis only (see Fig. 4) †Estimates from North America and Europe have been identified from systematic reviews or literature reviews where available ‡ For type 1 diabetes, the estimates identified are based on whole population § For Graves' disease estimates are based on hyperthyroidism cases.

RA and autoimmune thyroid diseases were by far the most common of the autoimmune conditions in China, where their prevalence appears to be closer to estimates from North America and Europe (Table 2) [[8], [74], [75], [76], [77]], particularly for GD [8,76]. Indeed, the highest estimates for RA and GD are higher than the highest estimates in North America and Europe, although this comes with important caveats. Specifically, we identified clear methodological, diagnostic and quality issues affecting studies examining the burden of these three conditions; the estimates were highly variable, and there were large differences between the fixed-effects and random-effects estimates, reflecting the number of small community-based surveys used to identify cases. For example, for RA, Langley et al. [53] reported a much higher prevalence estimate (2653 per 100,000 persons) than in other studies in which prevalence ranged from 52 to 761 per 100,000. This likely reflects the study's dependence on self-reported diagnoses obtained from an internet-based survey in a relatively small urban population (*n* = 13,307) [53], with much lower estimates (e.g. 98 per 100,000) derived from studies utilising large routine healthcare datasets with several million participants [35,47]. As such, case ascertainment methods are likely to contribute to some of the differences between and within conditions, as well as posing a challenge to identifying and interpreting regional differences. In contrast with studies in mainland China, all included studies from Taiwan and Hong Kong were based on large population-wide insurance databases, with or without active surveillance or registries.

Utilisation of routine healthcare data to study the epidemiology of autoimmune thyroid diseases presents challenges since the variable and frequently non-specific clinical presentation may result in incomplete capture of these conditions [76]. As such, most studies included in this review identified affected individuals using a combination of antibody levels, thyroid hormone levels and ultrasound scans in population based surveys, thereby identifying both diagnosed and undiagnosed cases. In addition, the burden of disease is strongly influenced by iodine intake, which was an exposure of interest in two included studies [36,62]. These gave conflicting findings across iodine deficient, adequate and excessive intake, suggesting a non-linear relationship with thyroid autoimmunity and the role of other factors. Many studies, including in Denmark and Slovenia, have hypothesised that iodine supplementation (through universal salt iodization policies) can accelerate the development of AT, particularly in initially deficient populations [[78], [79], [80], [81]].

Applying our fixed-effects and random-effects pooled estimates of the prevalence of the eight autoimmune diseases studied to the 2021 Chinese adult (15+) population [82], we generated two estimates of the proportion and number of adults affected (**Supplementary Table 5**). Our findings suggest that 31.5–35.5 million adults in China (2.7–3.0% of the Chinese population) have one of the eight most-common autoimmune diseases, which is broadly comparable to estimates from Western countries [6,7]. The vast majority of this burden in China is from RA and autoimmune thyroid diseases.

To the best of our knowledge, this is the first systematic review to consider the epidemiology of multiple autoimmune conditions in the Chinese population. Strengths of our study include the broad approach and search strategy used, which included both English and Chinese language studies, identifying a large number of estimates and cases across several regions in China, and allowing comparisons between and within autoimmune conditions, as well as with other global populations. In addition to the methodological issues outlined above, there are a number of limitations. First, we predominantly used crude incidence and prevalence rates to generate the pooled estimates, since insufficient studies presented standardised estimates. As such, confounding (e.g., by age and sex) may limit the validity of comparisons between the four locations studied and with global autoimmune disease prevalence and incidence estimates. For example, the demographic distribution in Hong Kong has a large surplus of middle-aged women, whereas mainland China has a slight surplus of young men. Second, there was considerable heterogeneity; most of our estimates generated an I^2^ above 90%, although this should be interpreted with caution given that I^2^ is a relative measure and may be biased when the number of studies is small (e.g. less than five). The potential for inaccurate estimation of between-study heterogeneity is seen for some of the sub-categories where two entirely overlapping estimates give an I^2^ of 0% (e.g. Fig. 1). Third, the studies and estimates identified were not necessarily representative of the adult population in China, and data were not available to generate population representative estimates, thus, limiting interpretation of the pooled estimates and population burden. Lastly, though the funnel plots to do not suggest it, publication bias is possible, particularly as smaller studies may have only been available through Chinese databases.

In conclusion, we find the burden of autoimmune diseases is generally lower in China than in other global populations, particularly in Europe and North America, although, for several of the most common conditions (autoimmune thyroid diseases and RA) rates are more comparable. Based on our findings, we estimate that up to 3 in every 100 Chinese adults (>31 million people) has an autoimmune condition. This is of concern for population health in China; autoimmune diseases are lifelong conditions associated with disability, co-morbidity and high healthcare costs. Our approach highlights the value of considering these conditions as a group of chronic and non-communicable diseases, particularly given the considerable associated burden of disease. However, given the marked variation between and within conditions in our study, as well as differences in study design, data sources and diagnosis, there is opportunity to better understand the true burden of autoimmune diseases in the Chinese population and contributing aetiologic factors through large-scale, robust and reliable epidemiological studies.

## Funding

Medical Research Council, United Kingdom.

## Key messages

• Autoimmune diseases account for a significant burden of disease in Europe and North America, where they are estimated to affect 3–9% of the population, but their epidemiology in other regions remains under-researched
• To the best of our knowledge, this is the first systematic review and meta-analysis to consider the burden of multiple autoimmune conditions in the adult Chinese population. We estimate that these conditions may affect 2.7–3.0% of the population (>31 million people).
• There is potential to understand the burden and shared aetiology of autoimmune diseases as a group of non-communicable and chronic conditions, through comparisons across conditions and across populations, though this requires large-scale, robust and reliable epidemiological studies with sufficient data for comparisons.

## CRediT authorship contribution statement

**Olaa Mohamed-Ahmed:** Conceptualization, Formal analysis, Investigation, Methodology, Project administration, Validation, Visualization, Writing – original draft, Writing – review & editing. **Lianhan Shang:** Methodology, Validation, Writing – review & editing, Investigation. **Lin Wang:** Investigation, Methodology, Validation, Writing – review & editing. **Zhengming Chen:** Conceptualization, Funding acquisition, Supervision, Validation, Writing – review & editing. **Christiana Kartsonaki:** Conceptualization, Investigation, Methodology, Supervision, Validation, Writing – review & editing. **Fiona Bragg:** Conceptualization, Funding acquisition, Investigation, Methodology, Project administration, Supervision, Validation, Writing – review & editing.

## Declaration of competing interest

We declare no competing interests.

## Data Availability

The data has been included in the supplementary files and can be made available on request.

## Appendix A Supplementary data

Supplementary material Image 1
