# Supplementary material for: Incidence and prevalence of autoimmune diseases in China: A systematic review and meta-analysis of epidemiological studies
Source: Glob Epidemiol. 2024 Aug 9;8:100158. doi: 10.1016/j.gloepi.2024.100158 (PMC11404186; doi:10.1016/j.gloepi.2024.100158)
Supplement: Supplementary file 1 — Supplementary material [file mmc1.docx]

**Supplementary Table 1. Search strategy**

1. incidence.mp. or Incidence/
2. Prevalence/ or prevalence.mp.
3. Epidemiologic Studies/ or Epidemiology/ or epidemiolog*.mp.
4. burden.mp.
5. registry.mp. or Registries/
6. china.mp. or China/
7. taiwan.mp. or Taiwan/
8. macau.mp. or Macau/
9. hong kong.mp. or Hong Kong/
10. autoimmune disease.mp. or Autoimmune Diseases/
11. autoimmune thyroiditis.mp. or Thyroiditis, Autoimmune/
12. hashimoto thyroiditis.mp. or Hashimoto Disease/
13. Graves Disease/ or autoimmune hypothyroidism.mp.
14. type 1 diabetes.mp. or Diabetes Mellitus, Type 1/
15. insulin dependent diabetes.mp.
16. multiple sclerosis.mp. or Multiple Sclerosis/
17. inflammatory bowel disease.mp. or Inflammatory Bowel Diseases/
18. ulcerative colitis.mp. or Colitis, Ulcerative/
19. crohn disease.mp. or Crohn Disease/
20. rheumatoid arthritis.mp. or Arthritis, Rheumatoid/
21. Lupus Erythematosus, Discoid/ or Lupus Vasculitis, Central Nervous System/ or Lupus Erythematosus, Cutaneous/ or Lupus Nephritis/ or Lupus Erythematosus, Systemic/ or lupus.mp.
22. 1 or 2 or 3 or 4 or 5
23. 6 or 7 or 8 or 9
24. 10 or 11 or 12 or 13 or 14 or 15 or 16 or 17 or 18 or 19 or 20 or 21
25. 22 and 23 and 24
26. limit 25 to (humans and yr="1995 –2023" and "all adult (19 plus years)")

**Supplementary Table 2. Full characteristics of included studies and their incidence estimates, by autoimmune disease (n=91)**

|  | Study location (Region) | Study location (Sub-regional) | Study period | Language | Data source | Included in meta-analysis? | Cases | Population | Crude incidence, per 100,000 persons (95% CI) | Standardised incidence,  per 100,000 persons (95% CI) | Mean or median age, years | Gender ratio (Female to Male) |
| --- | --- | --- | --- | --- | --- | --- | --- | --- | --- | --- | --- | --- |
| *Crohn's disease* | | | | | | | | | | | | |
| Chuang 2013 | Taiwan | Taiwan | 1998 - 2010 | English | RH | YES | 558 | 23,162,123 | 0.19 (0.18 - 0.20) | N/S | 37.9 | 0.47 |
| Jiang 2006 | Mainland China | Wuhan City | 1990 - 2003 | English | RH | YES | 63 | 6,000,000 | 0.07 (0.00 - 0.33) | N/S | 32.6 | 0.43 |
| Kuo 2015 | Taiwan | Taiwan | 2000 - 2010 | English | RH | NO ‡ ¶ | 526 | 253,528,860 | 0.21 (0.19 - 0.23) † | N/S | N/S | 0.45 |
| Leong 2004 | Hong Kong | Shatin | N/S | English | RH | NO ‡ | 80 | 956,323 | 1.00 (0.80 - 1.10) | 3.00 (2.30 - 3.70) | 33.0 | 0.40 |
| Lok 2007 | Hong Kong | Hong Kong | 1991 - 2006 | English | RH | NO ‡ | 27 | 1,095,400 | 0.25 (0.00 - 1.18) | N/S | 26.0 | 0.69 |
| Ng 2016 | Hong Kong | Hong Kong | 1981 - 2014 | English | AS/R | YES | 983 | 7,241,700 | 0.40 (0.00 – 1.25) | 1.46 (1.29 - 1.65)* | 30.0 | 0.54 |
| Ng 2019 | Mainland China | Guangzhou | 2011 - 2013 | English | AS/R | YES | 38 | 2,800,000 | 1.36 (0.96 - 1.86) | N/S | 33.0 | N/S |
| Ng 2019 | Hong Kong | Hong Kong | 2011 - 2013 | English | AS/R | NO ‡ | 81 | 6,400,000 | 1.27 (1.01 - 1.57) | N/S | 33.0 | N/S |
| Ng 2019 | Macau | Macau | 2011 - 2013 | English | AS/R | YES | 8 | 1,000,000 | 0.80 (0.35 - 1.58) | N/S | 33.0 | N/S |
| Ng 2019 | Mainland China | Wuhan | 2011 - 2013 | English | AS/R | NO § | 34 | 6,085,556 | 0.56 (0.37 - 0.75) | N/S | 33.0 | N/S |
| Ng 2019 | Mainland China | Xiangshan | 2012 - 2013 | English | AS/R | YES | 3 | 750,000 | 0.40 (0.08 - 1.17) | N/S | 33.0 | N/S |
| Ng 2019 | Combined | Various | 2011 - 2013 | English | AS/R | NO § | 323 | 83,136,305 | 0.36 (0.28 - 0.46) | N/S | 33.0 | N/S |
| Ng 2019 | Taiwan | Taiwan | 2011 - 2013 | English | AS/R | YES | 142 | 46,463,885 | 0.31 (0.26 - 0.36) | N/S | 33.0 | N/S |
| Ng 2019 | Mainland China | Daqing | 2012 - 2013 | English | AS/R | NO § | 2 | 1,343,363 | 0.15 (0.02 - 0.54) | N/S | 33.0 | N/S |
| Ng 2019 | Mainland China | Chengdu | 2011 - 2012 | English | AS/R | YES | 4 | 2,770,000 | 0.14 (0.04 - 0.37) | N/S | 33.0 | N/S |
| Ng 2019 | Mainland China | Kunming | 2011 - 2013 | English | AS/R | YES | 5 | 6,579,000 | 0.08 (0.02 - 0.18) | N/S | 33.0 | N/S |
| Ng 2019 | Mainland China | Xian | 2011 - 2012 | English | AS/R | YES | 6 | 8,944,500 | 0.07 (0.02 - 0.15) | N/S | 33.0 | N/S |
| Wei 2013 | Taiwan | Taiwan | 1998 - 2008 | English | RH | NO ‡ | 385 | N/S | 0.24 | N/S | 37.8 | 0.45 |
| Xu 2023 | Mainland China | Various | 2012 - 2016 | English | RH | NO ¶ | 9,746 | 513,300,000 | 0.71 (0.33 – 1.23) † | N/S | 37.1 | 0.84 |
| Yang 2014 | Mainland China | Daqing | 2012 - 2013 | English | AS/R | YES | 2 | 1,343,364 | 0.15 (0.02 - 0.54) | 0.13 (0.02 - 0.47) | 39.5 | N/S |
| Yang 2022 | Mainland China | Various | 2013 – 2016 | English | RH | NO ‡ | 4,817 | 150,617,725 | 0.93 (0.84 – 1.02) | N/S | N/S | 0.74 |
| Zeng 2013 | Mainland China | Zhongshan | 2011 - 2012 | English | AS/R | YES | 17 | 1,392,727 | 1.22 (0.64 - 1.80) | 1.09 | 25.0 | 0.42 |
| Zhao 2013 | Mainland China | Wuhan | 2010 - 2010 | English | AS/R | YES | 34 | 6,085,556 | 0.56 (0.37 - 0.75) | 0.51 (0.33 - 0.68) | 36.0 | 1.06 |
| *Ulcerative colitis* | | | | | | | | | | | | |
| Chow 2009 | Hong Kong | Shatin | 1985 - 2006 | English | RH | NO ‡ | 172 | 607,544 | N/S | 2.10 (1.10 - 3.70) | 48.4 | N/S |
| Chuang 2013 | Taiwan | Taiwan | 1998 - 2010 | English | RH | YES | 2,357 | 23,162,123 | 0.80 (0.77 - 0.84) | N/S | 44.7 | 0.67 |
| Jiang 2006 | Mainland China | Wuhan City | 1990 - 2003 | English | RH | YES | 389 | 6,000,000 | 0.46 (0.00 - 1.11) | N/S | 42.0 | 0.65 |
| Kuo 2015 | Taiwan | Taiwan | 2000 - 2010 | English | RH | NO ‡ ¶ | 2,125 | 253,519,007 | 0.84 (0.80 - 0.87) † | N/S | N/S | 0.65 |
| Lok 2008 | Hong Kong | Hong Kong | 1990 - 2006 | English | RH | NO ‡ | 73 | 1,095,400 | 0.37 (0.00 - 1.90) | 0.40 | 40.6 | 0.53 |
| Ng 2016 | Hong Kong | Hong Kong | 1981 - 2014 | English | AS/R | YES | 1,541 | 7,241,700 | 0.63 (0.00 - 1.69) | 1.51 (1.35 - 1.69)* | 41.0 | 0.79 |
| Ng 2019 | Mainland China | Guangzhou | 2011 - 2013 | English | AS/R | YES | 64 | 2,800,000 | 2.29 (1.76 - 2.92) | N/S | 43.0 | N/S |
| Ng 2019 | Mainland China | Daqing | 2012 - 2013 | English | AS/R | NO § | 25 | 1,343,363 | 1.86 (1.20 - 2.75) | N/S | 43.0 | N/S |
| Ng 2019 | Hong Kong | Hong Kong | 2011 - 2013 | English | AS/R | NO ‡ | 110 | 6,400,000 | 1.72 (1.41 - 2.07) | N/S | 43.0 | N/S |
| Ng 2019 | Macau | Macau | 2011 - 2013 | English | AS/R | YES | 16 | 1,000,000 | 1.60 (0.91 - 2.60) | N/S | 43.0 | N/S |
| Ng 2019 | Mainland China | Wuhan | 2011 - 2013 | English | AS/R | YES | 97 | 6,085,556 | 1.59 (1.28 - 1.91) | N/S | 43.0 | N/S |
| Ng 2019 | Mainland China | Kunming | 2011 - 2013 | English | AS/R | YES | 78 | 6,579,000 | 1.19 (0.94 - 1.48) | N/S | 43.0 | N/S |
| Ng 2019 | Mainland China | Xiangshan | 2012 - 2013 | English | AS/R | YES | 8 | 750,000 | 1.07 (0.46 - 2.10) | N/S | 43.0 | N/S |
| Ng 2019 | Taiwan | Taiwan | 2011 - 2013 | English | AS/R | YES | 329 | 46,463,885 | 0.71 (0.63 - 0.79) | N/S | 43.0 | N/S |
| Ng 2019 | Combined | Various | 2011 - 2013 | English | AS/R | NO § | 760 | 83,136,305 | 0.49 (0.32 - 0.74) | N/S | 43.0 | N/S |
| Ng 2019 | Mainland China | Xian | 2011 - 2012 | English | AS/R | YES | 42 | 8,944,500 | 0.47 (0.34 - 0.63) | N/S | 43.0 | N/S |
| Ng 2019 | Mainland China | Chengdu | 2011 - 2012 | English | AS/R | YES | 12 | 2,770,000 | 0.43 (0.22 - 0.76) | N/S | 43.0 | N/S |
| Shi 2016 | Hong Kong | Hong Kong | 1981 - 2013 | English | AS/R | NO ‡ | 1,225 | 7,200,000 | 1.30 (0.35 - 2.25) | N/S | 41.0 | 0.78 |
| Wei 2013 | Taiwan | Taiwan | 1998 - 2008 | English | RH | NO ‡ | 1,206 | N/S | 0.94 | N/S | 44.5 | 0.61 |
| Xu 2023 | Mainland China | Various | 2012 – 2016 | English | RH | NO ¶ | 84,402 | 513,300,000 | 8.95 (6.11 – 12.34) † | N/S | 47.2 | 0.85 |
| Yang 2014 | Mainland China | Daqing | 2012 - 2013 | English | AS/R | YES | 25 | 1,343,364 | 1.86 (1.20 - 2.75) | 1.64 (1.06 - 2.43) | 48.9 | 1.04 |
| Yang 2022 | Mainland China | Various | 2013 – 2016 | English | RH | NO ‡ | 23,178 | 150,617,725 | 4.45 (4.25 – 4.65) | N/S | N/S | 0.94 |
| Zeng 2013 | Mainland China | Zhongshan | 2011 - 2012 | English | AS/R | YES | 31 | 1,392,727 | 2.22 (2.02 - 2.27) | 2.05 | 38.0 | 0.63 |
| Zhao 2013 | Mainland China | Wuhan | 2010 - 2010 | English | AS/R | YES | 97 | 6,085,556 | 1.59 (1.28 - 1.91) | 1.45 (1.16 - 1.75) | 41.0 | 1.27 |
| *Inflammatory bowel disease* | | | | | | | | | | | | |
| Ng 2016 | Hong Kong | Hong Kong | 1981 - 2014 | English | AS/R | YES | 2,575 | 7,241,700 | 1.05 (0.00 – 2.42) | 3.12 (2.88 - 3.38) | 37.0 | 0.70 |
| Ng 2019 | Mainland China | Guangzhou | 2011 - 2013 | English | AS/R | YES | 102 | 2,800,000 | 3.64 (2.97 - 4.42) | N/S | N/S | N/S |
| Ng 2019 | Hong Kong | Hong Kong | 2011 - 2013 | English | AS/R | NO ‡ | 191 | 6,400,000 | 2.98 (2.58 - 3.44) | N/S | N/S | N/S |
| Ng 2019 | Macau | Macau | 2011 - 2013 | English | AS/R | YES | 24 | 1,000,000 | 2.40 (1.54 - 3.57) | N/S | N/S | N/S |
| Ng 2019 | Mainland China | Wuhan | 2011 - 2013 | English | AS/R | NO § | 131 | 6,085,556 | 2.15 (1.78 - 2.52) | N/S | N/S | N/S |
| Ng 2019 | Mainland China | Daqing | 2012 - 2013 | English | AS/R | NO § | 27 | 1,343,364 | 2.01 (1.32 - 2.92) | N/S | N/S | N/S |
| Ng 2019 | Mainland China | Xiangshan | 2012 - 2013 | English | AS/R | YES | 11 | 750,000 | 1.47 (0.73 - 2.62) | N/S | N/S | N/S |
| Ng 2019 | Mainland China | Kunming | 2011 - 2013 | English | AS/R | YES | 83 | 6,579,000 | 1.26 (1.00 - 1.56) | N/S | N/S | N/S |
| Ng 2019 | Taiwan | Taiwan | 2011 - 2013 | English | AS/R | YES | 471 | 46,463,885 | 1.01 (0.92 - 1.11) | N/S | N/S | N/S |
| Ng 2019 | Combined | Various | 2011 - 2013 | English | AS/R | NO § | 1,104 | 83,136,305 | 0.80 (0.57 - 1.11) | N/S | N/S | N/S |
| Ng 2019 | Mainland China | Chengdu | 2011 - 2012 | English | AS/R | YES | 16 | 2,770,000 | 0.58 (0.33 - 0.94) | N/S | N/S | N/S |
| Ng 2019 | Mainland China | Xian | 2011 - 2012 | English | AS/R | YES | 48 | 8,944,500 | 0.54 (0.40 - 0.71) | N/S | N/S | N/S |
| Xu 2023 | Mainland China | Various | 2012 – 2016 | English | RH | NO ¶ | 95,555 | 513,300,000 | 10.04 (6.95 – 13.71) † | 10.76 (10.65 – 10.87) † | 46.2 | 0.86 |
| Yang 2014 | Mainland China | Daqing | 2012 - 2013 | English | AS/R | YES | 27 | 1,343,364 | 2.01 (1.32 - 2.92) | 1.77 (1.16 - 2.59) | 48.2 | 0.90 |
| Zeng 2013 | Mainland China | Zhongshan | 2011 - 2012 | English | AS/R | YES | 48 | 1,392,727 | 3.44 (2.47 - 4.42) | 3.14 | N/S | 0.55 |
| Zhao 2013 | Mainland China | Wuhan | 2010 - 2010 | English | AS/R | YES | 131 | 6,085,556 | 2.15 (1.78 - 2.52) | 1.96 (1.62 - 2.30) | 40.0 | 1.21 |
| *Multiple sclerosis* | | | | | | | | | | | | |
| Hsu 2021 | Taiwan | Taiwan | 2007 - 2016 | English | AS/R | YES | 555 | 23,600,000 | 0.31 (0.11 – 0.50) | N/S | 36.9 | 2.91 |
| Lai 2009 | Taiwan | Taiwan | 2000 - 2005 | English | RH | YES | 888 | 22,770,000 | 0.79 (0.48 - 1.10) | N/S | N/S | 3.50 |
| Liao 2022 | Taiwan | Taiwan | 2001 – 2015 | English | AS/R | NO ‡ | 1,553 | 23,737,000 | 0.46 (0.13 – 0.78) | 0.42 | N/S | 3.11 |
| Tian 2020 | Mainland China | Various | 2016 - 2018 | English | RH | YES | 9,879 | 1,200,000,000 | 0.27 (0.25 – 0.29) | 0.29 (0.28 - 0.29) | 45.3 | 2.02 |
| *Type 1 diabetes* | | | | | | | | | | | | |
| Jiang 2012 | Taiwan | Taiwan | 2000 - 2009 | English | RH | NO ‡ | 8,043 | 23,120,000 | 2.73 (1.97 - 3.49) | 2.98 | N/S | N/S |
| Lin 2014 | Taiwan | Taiwan | 1999 - 2010 | English | RH | NO ‡ | 7,225 | 23,000,000 | 3.34 | 3.34 | N/S | N/S |
| Liu 2021 | Mainland China | Beijing | 2008 - 2017 | English | AS/R | NO ¶ | 5,914 | 21,700,000 | 3.10 (2.41 - 3.79) † | N/S | N/S | 0.88 |
| Luk 2020 | Hong Kong | Hong Kong | 2002 - 2015 | English | AS/R | YES | 2,426 | 7,291,300 | 2.49 (1.17 - 3.81) | N/S | 32.5 | 0.95 |
| Sheen 2019 | Taiwan | Taiwan | 2005 - 2014 | English | RH | YES | 4,931 | 23,127,300 | 2.13 (1.54 - 2.73) | 2.61 | N/S | 1.22 |
| Weng 2018 | Mainland China | Various | 2010 - 2013 | English | AS/R | NO ¶ | 5,018 | 135,408,192 | 1.01 (0.18 - 1.84) † | N/S | N/S | 0.85 |
| *Systemic lupus erythematosus* | | | | | | | | | | | | |
| Chiu 2010 | Taiwan | Taiwan | 2000 - 2007 | English | RH | NO ‡ | 22,182 | 23,000,000 | 8.10 (6.83 - 9.37) | N/S | N/S | 7.10 |
| Lai 2023 | Mainland China | Ningbo | 2016 – 2021 | Chinese | RH | NO ¶ | 451 | 1,551,921 | 8.14 (7.41 – 8.93) † | N/S | 46.7 | 3.36 |
| Leong 2021 | Taiwan | Taiwan | 2001 – 2011 | English | AS/R | NO ‡ ¶ | 667 | 7,649,831 | 8.7 (8.1 – 9.4) † | N/S | N/S | 7.28 |
| Lin 2012 | Taiwan | Taiwan | 2000 - 2008 | English | RH | NO ‡ | 2,721 | 877,959 | 3.66 (0.00 - 15.31) | N/S | N/S | N/S |
| Mok 2008 | Hong Kong | Hong Kong | 2000 - 2006 | English | AS/R | YES | 442 | 1,000,000 | 3.10 (0.00 - 7.22) | N/S | 32.3 | 9.80 |
| See 2013 | Taiwan | Taiwan | 2005 - 2009 | English | RH | NO ‡ | 435 | 1,000,000 | 7.20 (6.50 - 8.00) | N/S | N/S | 6.30 |
| Yeh 2013 | Taiwan | Taiwan | 2003 - 2008 | English | RH | YES | 6,675 | 22,600,000 | 4.87 (4.16 - 5.58) | N/S | N/S | 7.15 |
| Yu 2013 | Taiwan | Taiwan | 2000 - 2008 | English | RH | NO ¶ | 671 | 8,026,058 | 8.40 (7.70 - 9.00) † | N/S | N/S | 8.00 |
| *Rheumatoid arthritis* | | | | | | | | | | | | |
| Kuo 2013 | Taiwan | Taiwan | 2002 - 2007 | English | RH | YES | 15,967 | 23,000,000 | 15.80  (14.72 - 16.88) | N/S | 53.7 | 3.50 |
| Lai 2012 | Taiwan | Taiwan | 2000 - 2007 | English | RH | NO ‡ | 40,995 | 18,600,000 | 22.10  (19.97 - 24.23) | 15.80 | N/S | 3.20 |
| See 2013 | Taiwan | Taiwan | 2005 - 2009 | English | RH | NO ‡ | 1,016 | 1,000,000 | 17.20  (16.10 - 18.40) | N/S | N/S | 3.90 |
| Yu 2013 | Taiwan | Taiwan | 2000 - 2008 | English | RH | NO ‡ ¶ | 1,390 | 8,026,058 | 17.30  (16.40 - 18.20) † | N/S | N/S | 3.00 |
| *Graves' Disease* | | | | | | | | | | | | |
| Teng 2006 | Mainland China | Panshan (ID) | 1999 - 2004 | English | S | NO ¶ | 7 | 884 | 158.4 (0.0 – 745.0) | N/S | N/S | N/S |
| Teng 2006 | Mainland China | Huanghua (IE) | 1999 - 2004 | English | S | NO ¶ | 5 | 864 | 115.7 (0.0 – 623.0) | N/S | N/S | N/S |
| Teng 2006 | Mainland China | Zhangwu (IA) | 1999 - 2004 | English | S | NO ¶ | 7 | 1,270 | 110.2 (0.0 – 518.6) | N/S | N/S | N/S |
| *Autoimmune thyroiditis* | | | | | | | | | | | | |
| Teng 2006 | Mainland China | Huanghua (IE) | 1999 - 2004 | English | S | NO ¶ | 11 | 864 | 254.6 (0.0 – 1007.0) | N/S | N/S | N/S |
| Teng 2006 | Mainland China | Zhangwu (IA) | 1999 - 2004 | English | S | NO ¶ | 13 | 1,270 | 204.7 (0.0 – 761.2) | N/S | N/S | N/S |
| Teng 2006 | Mainland China | Panshan (ID) | 1999 - 2004 | English | S | NO ¶ | 2 | 884 | 45.3 (0.0 – 358.8) | N/S | N/S | N/S |

† per 100,000 person-years; ‡ excluded due to partial or complete overlap of data source; § excluded due to duplicate data ¶ excluded due to insufficient information; * cumulative incidence

(ID) = iodine deficient, (IA) = iodine adequate, (IE) = iodine excessive, (Z) = Zhuang ethnicity, (H) = Han ethnicity

AS/R = Active surveillance or registry, RH = Routine healthcare, S = Survey

**Supplementary Table 3. Full characteristics of included studies and their prevalence estimates, by autoimmune disease (n=70)**

|  | Study location (Region) | Study location (Sub-national) | Study period | Language | Data source | Included in meta-analysis? | Cases | Population | Crude prevalence, per 100,000 persons (95% CI) | Standardised prevalence,  per 100,000 persons  (95% CI) | Mean or median age, years | Gender ratio (Female to Male) |  |
| --- | --- | --- | --- | --- | --- | --- | --- | --- | --- | --- | --- | --- | --- |
| *Crohn's disease* | | | | | | | | | | | | | |
| Chuang 2013 | Taiwan | Taiwan | 1998 - 2010 | English | RH | YES | 479 | 23,162,123 | 2.05 (1.87 – 2.23) | 2.05 | 37.9 | 0.5 |  |
| Jiang 2006 | Mainland China | Wuhan City | 1990 - 2003 | English | RH | YES | 63 | 6,000,000 | 1.05 (0.79 – 1.31) | N/S | 32.6 | N/S |  |
| Kuo 2015 | Taiwan | Taiwan | 2000 - 2010 | English | RH | NO ‡ ¶ | 526 | 253,528,860 | 1.42 (1.37 - 1.47) † | N/S | N/S | 0.5 |  |
| Lok 2007 | Hong Kong | Hong Kong | 1991 - 2006 | English | RH | NO ‡ | 27 | 1,095,400 | 2.70 (1.73 - 3.67) | N/S | 26.0 | 0.7 |  |
| Ng 2016 | Hong Kong | Hong Kong | 1981 - 2014 | English | AS/R | YES | 983 | 7,241,700 | 17.49 (16.39 - 18.58) | 18.63 (17.44 - 19.88) | 30.0 | 0.5 |  |
| Wei 2013 | Taiwan | Taiwan | 1998 - 2008 | English | RH | NO ‡ | 385 | N/S | 1.8 | N/S | 37.8 | 0.5 |  |
| Yang 2022 | Mainland China | Various | 2016 - 2016 | English | RH | YES | 5,281 | 150,617,725 | 3.5 (3.4 – 3.5) | 3.39 (3.29 – 3.48) | N/S | 0.7 | |
| *Ulcerative colitis* | | | | | | | | | | | | | |
| Chow 2009 | Hong Kong | Shatin | 1985 - 2006 | English | RH | NO ‡ | 172 | 607,544 | 26.50 (22.60 - 30.90) | N/S | 48.4 | N/S |  |
| Chuang 2013 | Taiwan | Taiwan | 1998 - 2010 | English | RH | YES | 2,065 | 23,162,123 | 8.49 (8.11 – 8.87) | 8.5 | 44.7 | 0.7 |  |
| Jiang 2006 | Mainland China | Wuhan City | 1990 - 2003 | English | RH | YES | 389 | 6,000,000 | 6.48 (5.84 – 7.12) | N/S | 42.0 | N/S |  |
| Kuo 2015 | Taiwan | Taiwan | 2000 - 2010 | English | RH | NO ‡ ¶ | 2,125 | 253,519,007 | 6.19 (6.09 - 6.29) † | N/S | N/S | 0.7 |  |
| Lok 2008 | Hong Kong | Hong Kong | 1990 - 2006 | English | RH | NO ‡ | 73 | 1,095,400 | 6.30 (4.81 - 7.78) | 7.0 | 40.6 | 0.5 |  |
| Ng 2016 | Hong Kong | Hong Kong | 1981 - 2014 | English | AS/R | YES | 1,541 | 7,241,700 | 27.41 (26.04 - 28.78) | 24.51 (23.26 - 25.81) | 41.0 | 0.8 |  |
| Wei 2012 | Taiwan | Taiwan | 1988 - 2008 | English | RH | NO ‡ | 406 | N/S | 7.40 | N/S | 36.0 | 0.7 |  |
| Wei 2013 | Taiwan | Taiwan | 1998 - 2008 | English | RH | NO ‡ | 1,206 | N/S | 7.62 | N/S | 44.5 | 0.6 |  |
| Yang 2022 | Mainland China | Various | 2016 - 2016 | English | RH | YES | 24,989 | 150,617,725 | 16.6 (16.4 – 16.8) | 17.2 (17.0 – 17.5) | N/S | 1.0 | |
| *Inflammatory bowel disease* | | | | | | | | | | | | | |
| Chen 2008 | Taiwan | Taiwan | 2004 - 2004 | English | RH | YES | 1,206 | 21,422,317 | 5.60 (5.28 - 5.92) | N/S | N/S | 0.5 |  |
| Ng 2016 | Hong Kong | Hong Kong | 1981 - 2014 | English | AS/R | YES | 2,575 | 7,241,700 | 45.81 (44.04 - 47.58) | 43.99 (42.25 - 45.80) | 37.0 | 0.7 |  |
| *Multiple sclerosis* | | | | | | | | | | | | | |
| Cheng 2007 | Mainland China | Shanghai | 2004 - 2004 | English | AS/R | YES | 123 | 8,860,000 | 1.39 (1.16 - 1.66) | N/S | 46.1 | 1.8 |  |
| Lai 2009 | Taiwan | Taiwan | 2000 - 2005 | English | RH | YES | 674 | 22,770,000 | 2.96 (2.74 - 3.18) | N/S | N/S | 3.5 |  |
| Lau 2002 | Hong Kong | Hong Kong | 1999 - 1999 | English | AS/R | NO ‡ | 53 | 6,800,000 | 0.77 (0.56 – 0.98) | N/S | 32.8 | 9.6 |  |
| Lau 2008 | Hong Kong | Hong Kong | 1996 - 2006 | English | RH | YES | 106 | 2,200,000 | 4.80 (3.88 - 5.72) | N/S | 43.9 | 3.2 |  |
| Liao 2022 | Taiwan | Taiwan | 2015 – 2015 | English | AS/R | YES | 1,761 | 23,737,000 | 7.42 (7.07 – 7.77) | N/S | N/S | 3.5 | |
| Liu 2022 | Mainland China | Guangzhou | 2021 - 2021 | English | RH | YES | 143 | 18,676,605 | 0.77 (0.65 – 0.90) | 0.92 (0.77 – 1.10) | 30.0 | 2.3 | |
| Tsai 2004 | Taiwan | Taiwan | 1985 - 1999 | English | RH | YES | 429 | 22,405,000 | 1.90 (1.72 – 2.08) | N/S | 29.9 | 5.0 |  |
| Xu 2021 | Mainland China | Various (urban) | 2012 - 2016 | English | AS/R | YES | 3,727 | 195,440,000 | 2.44 (2.18 - 2.72) | 2.29 (2.21 - 2.38) | 43.9 | 2.2 |  |
| *Type 1 diabetes* | | | | | | | | | | | | | |
| Jiang 2012 | Taiwan | Taiwan | 2000 - 2009 | English | RH | NO ‡ | 8,043 | 23,120,000 | 26.9 (26.2 - 27.6) | N/S | N/S | N/S |  |
| Sheen 2019 | Taiwan | Taiwan | 2005 - 2014 | English | RH | YES | 11,225 | 23,434,000 | 47.9 (47.0 - 48.8) | 50.0 | N/S | 1.2 |  |
| *Systemic lupus erythematosus* | | | | | | | | | | | | | |
| Chiu 2010 | Taiwan | Taiwan | 2000 - 2007 | English | RH | NO ‡ | 22,182 | 23,000,000 | 55.7 (54.7 - 56.7) | N/S | N/S | 7.1 |  |
| Leong 2021 | Taiwan | Taiwan | 2011 – 2011 | English | AS/R | YES | 699 | 862,136 | 81.1 (75.3 – 87.3) | N/S | N/S | N/S | |
| Li 2012 | Mainland China | Beijing | N/S | English | S | YES | 3 | 10,556 | 28.4 (0.0 - 60.0) | N/S | N/S | N/S |  |
| Mok 2003 | Hong Kong | Hong Kong | 2001 - 2001 | English | RH | YES | 876 | 1,490,000 | 58.8 (54.9 - 62.7) | N/S | 30.1 | 9.3 |  |
| See 2013 | Taiwan | Taiwan | 2005 - 2009 | English | RH | NO ‡ | 435 | 1,000,000 | 43.5 (39.4 - 47.6) | N/S | N/S | 6.3 |  |
| Yeh 2013 | Taiwan | Taiwan | 2003 - 2008 | English | RH | YES | 22,035 | 22,600,000 | 97.5 (96.2 - 98.8) | N/S | N/S | 7.2 |  |
| Yu 2013 | Taiwan | Taiwan | 2000 - 2000 | English | RH | YES | 356 | 963,355 | 37.0 (10.0 - 41.0) | N/S | N/S | 7.8 |  |
| Zou 2014 | Mainland China | Anhui | 2009 - 2010 | English | S | YES | 471 | 1,253,832 | 37.6  (34.2 - 41.0) | 36.0  (35.5 - 36.5) | 39.1 | 11.0 |  |
| *Rheumatoid arthritis* | | | | | | | | | | | | | |
| Dai 2003 | Mainland China | Shanghai | 1997 - 1998 | English | S | YES | 31 | 6,584 | 470.8  (300.0 - 640.0) | 280.0  (150.0 - 410.0) | N/S | 3.2 |  |
| Kuo 2013 | Taiwan | Taiwan | 2002 - 2007 | English | RH | YES | 27,171 | 23,000,000 | 97.5  (96.2 - 98.8) | N/S | 53.7 | 3.5 |  |
| Lai 2012 | Taiwan | Taiwan | 2000 - 2007 | English | RH | NO ‡ | 40,995 | 18,600,000 | 146.1  (144.4 - 147.8) | 99.6 | N/S | 3.2 |  |
| Langley 2011 | Mainland China | Various (cities) | 2009 - 2009 | English | S | YES | 353 | 13,307 | 2652.7  (2379.7 - 2925.8) | N/S | N/S | N/S |  |
| Li 2012 | Mainland China | Beijing | N/S | English | S | YES | 43 | 10,556 | 407.4  (300.0 - 550.0) | 280.0  (190.0 - 410.0) | N/S | 6.0 |  |
| See 2013 | Taiwan | Taiwan | 2005 - 2009 | English | RH | NO ‡ | 1,016 | 1,000,000 | 101.6  (95.4 - 107.7) | N/S | N/S | 3.9 |  |
| Shi 2003 | Mainland China | Shanghai | 2001 - 2002 | Chinese | S | YES | 50 | 7,575 | 660.1  (477.7 - 842.5) | 520.0 | 48.0 | 1.9 |  |
| Sun 2013 | Mainland China | Henan | 2012 - 2012 | Chinese | S | YES | 63 | 8,274 | 761.4  (574.1 - 948.7) | 710.0 | 61.0 | 2.6 |  |
| Yu 2013 | Taiwan | Taiwan | 2000 - 2000 | English | RH | YES | 505 | 963,355 | 52.4  (14.2 - 57.2) | N/S | N/S | 4.4 |  |
| Zeng 1997 | Mainland China | Shantou | 1985 - 1995 | Chinese | S | YES | 47 | 22,049 | 213.0  (152.1 - 273.9) | N/S | 47.0 | N/S |  |
| Zeng 2007 | Mainland China | Nanning | N/S | Chinese | S | YES | 20 | 7,407 | 270.0  (151.8 - 388.2) | N/S | N/S | 3.1 |  |
| Zeng 2007 | Mainland China | Nanning | N/S | Chinese | S | YES | 19 | 6,826 | 278.3  (153.4 - 403.3) | N/S | N/S | 2.8 |  |
| Zeng 2015 | Mainland China | Shantou | 2012 - 2012 | English | S | YES | 19 | 4,056 | 468.4  (258.3 - 678.6) | 350.0  (170.0 - 530.0) | 48.4 | 2.1 |  |
| Zhang 1995 | Mainland China | Shantou | N/S | Chinese | S | YES | 14 | 5,057 | 320.0  (160.0 - 470.0) | N/S | 55.0 | 15.0 |  |
| Zhang 1995 | Mainland China | Beijing | N/S | Chinese | S | YES | 16 | 4,192 | 340.0  (200.0 - 510.0) | N/S | 44.0 | 15.0 |  |
| *Graves' disease* | | | | | | | | | | | | | |
| Li 2000 | Mainland China | Daqing | 1993 - 1995 | English | S | YES | 301 | 100,123 | 300.6  (266.7- 334.5) | N/S | N/S | 4.00 |  |
| Gu 2016 | Mainland China | Zhejiang | 2011 - 2011 | English | S | YES | 10 | 17,056 | 58.6  (22.3 - 95.0) | N/S | N/S | N/S |  |
| Shan 2016 | Mainland China | Various (cities) | 2011 - 2011 | English | S | YES | 92 | 15,008 | 613.0  (488.1 - 737.9) | N/S | 45.5 | N/S |  |
| Teng 2006 | Mainland China | Huanghua (IE) | 1999 - 1999 | English | S | YES | 12 | 1,074 | 1117.3  (488.7 - 1746.0) | N/S | N/S | N/S |  |
| Teng 2006 | Mainland China | Zhangwu (IA) | 1999 - 1999 | English | S | YES | 20 | 1,584 | 1262.6  (712.8 - 1812.5) | N/S | N/S | N/S |  |
| Teng 2006 | Mainland China | Panshan (ID) | 1999 - 1999 | English | S | YES | 15 | 1,103 | 1359.9  (676.4 - 2043.5) | N/S | N/S | N/S |  |
| Wan 2020 | Mainland China | Shandong (IA) | N/S | English | S | YES | 2 | 392 | 510.2  (0.0 - 1215.5) | N/S | N/S | 2.4 |  |
| Wan 2020 | Mainland China | Shandong (IE) | N/S | English | S | NO ¶ | - | 424 | 0.0  (0.0 - 0.0) | N/S | N/S | 2.1 |  |
| Wan 2020 | Mainland China | Shandong (ID) | N/S | English | S | YES | 2 | 409 | 489.0  (0.0 - 1165.1) | N/S | N/S | 4.0 |  |
| Wang 2021 | Mainland China | Various | 2015 - 2017 | English | S | YES | 404 | 78,470 | 515.0 (464.9 – 565.1) | N/S | 42.9 | 1.9 | |
| Wu 2015 | Mainland China | Shaanxi | 2013 - 2013 | English | S | YES | 37 | 6,152 | 600.0  (400.0 - 700.0) | N/S | N/S | N/S |  |
| *Autoimmune thyroiditis* | | | | | | | | | | | | | |
| Gu 2016 | Mainland China | Zhejiang | 2011 - 2011 | English | S | YES | 17 | 17,056 | 99.7  (52.3 - 147.0) | N/S | N/S | N/S |  |
| Teng 2006 | Mainland China | Panshan (ID) | 1999 - 1999 | English | S | YES | 5 | 1,103 | 453.3  (56.9 - 849.8) | N/S | N/S | N/S |  |
| Teng 2006 | Mainland China | Zhangwu (IA) | 1999 - 1999 | English | S | YES | 27 | 1,584 | 1704.5  (1067.1 - 2342.0) | N/S | N/S | N/S |  |
| Teng 2006 | Mainland China | Huanghua (IE) | 1999 - 1999 | English | S | YES | 30 | 1,074 | 2793.3  (1807.8 - 3778.8) | N/S | N/S | N/S |  |
| Wan 2020 | Mainland China | Shandong (IA) | N/S | English | S | YES | 13 | 392 | 3316.3  (1543.7 - 5089.0) | N/S | N/S | 2.4 |  |
| Wan 2020 | Mainland China | Shandong (IE) | N/S | English | S | YES | 14 | 424 | 3301.9  (1601.0 - 5002.7) | N/S | N/S | 2.1 |  |
| Wan 2020 | Mainland China | Shandong (ID) | N/S | English | S | YES | 22 | 409 | 5379.0  (3192.5 - 7565.4) | N/S | N/S | 4.0 |  |
| Wu 2015 | Mainland China | Shaanxi | 2013 - 2013 | English | S | YES | 172 | 6,152 | 2800.0  (2400.0 - 3300.0) | N/S | N/S | N/S |  |

† per 100,000 person-years; ‡ excluded due to partial or complete overlap of data source; § excluded due to duplicate data ¶ excluded due to insufficient information

(ID) = iodine deficient, (IA) = iodine adequate, (IE) = iodine excessive, (Z) = Zhuang ethnicity, (H) = Han ethnicity; AS/R = Active surveillance or registry, RH = Routine healthcare, S = Survey

| **Citation** | Autoimmune diseases | Was the sample frame appropriate to address the target population? | Were study participants sampled in an appropriate way? | Was the sample size adequate? | Were the study subjects and the setting described in detail? | Was the data analysis conducted with sufficient coverage of the identified sample? | Were valid methods used for the identification of the condition? | Was the condition measured in a standard reliable way for all participants? | Was there appropriate statistical analysis? | Was the response rate adequate, and if not, was the low response rate managed appropriately? |
| --- | --- | --- | --- | --- | --- | --- | --- | --- | --- | --- |
| Chen-2008 | IBD | Yes | Yes | Yes | Yes | Yes | Yes | Yes | Yes | Yes |
| Cheng-2007 | MS | Yes | Yes | Yes | Yes | Yes | Yes | Yes | Yes | Unsure |
| Chiu-2010 | SLE | Yes | Yes | Yes | Yes | Yes | Yes | Yes | Yes | Yes |
| Chow-2009 | UC | Yes | Yes | Yes | Yes | Yes | Yes | Yes | Yes | Yes |
| Chuang-2013 | CD, UC | Yes | Yes | Yes | Yes | Yes | Yes | Yes | Yes | Yes |
| Dai-2003 | RA | Yes | Yes | Yes | Yes | Yes | Yes | Yes | Yes | Yes |
| Gu-2016 | AT, GD | Yes | Yes | Unsure | Yes | Yes | Yes | Yes | Yes | Yes |
| Hsu-2021 | MS | Yes | Yes | Yes | Yes | Yes | Yes | Yes | Yes | Yes |
| Jiang-2006 | CD, UC | Unsure | Yes | Yes | No | Unsure | Yes | Yes | No | Yes |
| Jiang-2012 | T1D | Yes | Yes | Yes | Yes | Yes | Yes | Yes | Yes | Yes |
| Kuo-2015 | CD, UC | Yes | Yes | Yes | Yes | Yes | Yes | Yes | Yes | Yes |
| Kuo-2013 | RA | Yes | Yes | Yes | Yes | Yes | Yes | Yes | Yes | Yes |
| Lai-2023 | SLE | Yes | Yes | Yes | Yes | Yes | Yes | Yes | Yes | Yes |
| Lai-2012 | RA | Yes | Yes | Yes | Yes | Yes | Yes | Yes | Yes | Yes |
| Lai-2009 | MS | Yes | Yes | Yes | Yes | Yes | Yes | Yes | Yes | Yes |
| Langley-2011 | RA | Yes | Yes | Yes | Yes | Yes | No | Yes | Yes | Unsure |
| Lau-2002 | MS | Yes | Unsure | Yes | Yes | Yes | Yes | Unsure | Yes | Yes |
| Lau-2008 | MS | Yes | Unsure | Yes | Unsure | Unsure | Yes | Yes | Yes | Yes |
| Leong-2021 | SLE | Yes | Yes | Yes | Yes | Yes | Yes | Yes | Yes | Yes |
| Leong-2004 | CD | Yes | Yes | Yes | Yes | Yes | Yes | Yes | Yes | Yes |
| Li-2000 | GD | Yes | Yes | Yes | Yes | Yes | Yes | Yes | Yes | Unsure |
| Li-2012 | SLE, RA | Yes | Yes | Unsure | Yes | Yes | Yes | Yes | Yes | Yes |
| Liao-2022 | MS | Yes | Yes | Yes | Yes | Yes | Yes | Yes | Yes | Yes |
| Lin-2012 | SLE | Yes | Yes | Yes | Yes | Yes | Yes | Yes | Yes | Yes |
| Lin-2014 | T1D | Yes | Yes | Yes | Yes | Yes | Yes | Yes | Yes | Yes |
| Liu-2022 | MS | Yes | Yes | Yes | Yes | Yes | Yes | Yes | Yes | Yes |
| Liu-2021 | T1D | Yes | Yes | Yes | Yes | Yes | Yes | Yes | Yes | Yes |
| Lok-2007 | CD | Yes | Yes | Yes | Yes | Yes | Yes | Yes | Yes | Yes |
| Lok-2008 | UC | Yes | Yes | Yes | Yes | Yes | Yes | Yes | Yes | Yes |
| Luk-2020 | T1D | Yes | Yes | Yes | Yes | Yes | Yes | Yes | Yes | Yes |
| Mok-2003 | SLE | Yes | Yes | Yes | Yes | Yes | Yes | Yes | Yes | Yes |
| Mok-2008 | SLE | Yes | Yes | Yes | Yes | Yes | Yes | Yes | Yes | Yes |
| Ng-2016 | CD, UC, IBD | Yes | Yes | Yes | Yes | Yes | Yes | Yes | Yes | Yes |
| Ng-2019 | CD, UC, IBD | Yes | Yes | Yes | Yes | Yes | Yes | Yes | Yes | Yes |
| See-2013 | SLE, RA | Yes | Yes | Yes | Yes | Yes | Yes | Yes | Yes | Yes |
| Shan-2016 | GD | Yes | Yes | Yes | Yes | Yes | Yes | Yes | Yes | Unsure |
| Sheen-2019 | T1D | Yes | Yes | Yes | Yes | Yes | Unsure | Yes | Yes | Yes |
| Shi-2016 | UC | Yes | Yes | Yes | Yes | Yes | Yes | Yes | Yes | Yes |
| Shi-2003 | RA | No | Yes | Yes | Yes | Yes | Yes | Yes | Yes | Yes |
| Sun-2013 | RA | No | Yes | Yes | Yes | Yes | Yes | Yes | No | Yes |
| Teng-2006 | GD, AT | Yes | Yes | Unsure | Yes | Yes | Yes | Yes | Yes | No |
| Tian-2020 | MS | Yes | Yes | Yes | Yes | Yes | Yes | Yes | Yes | Yes |
| Tsai-2004 | MS | Yes | Yes | Yes | Yes | Unsure | Yes | Yes | Unsure | Yes |
| Wan-2020 | GD, AT | Yes | Yes | Yes | Yes | Yes | Yes | Yes | Yes | Unsure |
| Wang-2021 | GD | Yes | Yes | Yes | Yes | Yes | Yes | Yes | Yes | Yes |
| Wei-2013 | CD, UC | Yes | Yes | Yes | Yes | Yes | Yes | Yes | Yes | Yes |
| Wei-2012 | UC | Yes | Yes | Yes | Yes | Yes | Yes | Yes | Yes | Yes |
| Weng-2018 | T1D | Yes | Yes | Yes | Yes | Yes | Yes | Yes | Yes | Yes |
| Wu-2015 | GD, AT | Yes | Yes | Yes | Yes | Yes | Yes | Yes | Yes | Unsure |
| Xu-2023 | CD, UC, IBD | Yes | Yes | Yes | Yes | Yes | Yes | Yes | Yes | Yes |
| Xu-2021 | MS | Yes | Unsure | Yes | Yes | Yes | Yes | Yes | Yes | Yes |
| Yang-2022 | CD, UC | Yes | Yes | Yes | Yes | Yes | Yes | Yes | Yes | Yes |
| Yang-2014 | CD, UC, IBD | Yes | Yes | No | Yes | Yes | Yes | Yes | Yes | Yes |
| Yeh-2013 | SLE | Yes | Yes | Yes | Yes | Yes | Yes | Yes | Yes | Yes |
| Yu-2013 | SLE, RA | Yes | Yes | Yes | Yes | Yes | Yes | Yes | Yes | Yes |
| Zeng-2013 | CD, UC, IBD | Yes | Yes | Yes | Yes | Yes | Yes | Yes | Yes | Yes |
| Zeng-2015 | RA | Yes | Yes | Unsure | Unsure | Yes | Yes | Yes | Yes | Yes |
| Zeng-2007 | RA | No | Yes | Unsure | Yes | Yes | Yes | Yes | Yes | Yes |
| Zeng-1997 | RA | No | No | Unsure | Unsure | Unsure | Yes | No | Unsure | Yes |
| Zhang-1995 | RA | No | No | No | Unsure | Unsure | Yes | No | Unsure | Yes |
| Zhao-2013 | CD, UC, IBD | Yes | Yes | Yes | Yes | Yes | Yes | Yes | Yes | Yes |
| Zou-2014 | SLE | Yes | Yes | Yes | Yes | Yes | Yes | Yes | Yes | Yes |

**Supplementary Table 4. Quality assessment of included studies (n=62)**

AT = autoimmune thyroiditis, CD = Crohn’s disease, GD = Graves’ disease, IBD = inflammatory bowel disease, MS = multiple sclerosis, RA = rheumatoid arthritis, SLE = systemic lupus erythematosus, T1D = Type 1 diabetes, UC = ulcerative colitis

**Supplementary Table 5. Estimates of the number of cases of each autoimmune disease in the adult population of mainland China**

| Autoimmune disease | Estimate 1* | | | Estimate 2† | | |
| --- | --- | --- | --- | --- | --- | --- |
|  | **Fixed-effects pooled prevalence** | | **Expected cases** | **Random-effects pooled prevalence** | | **Expected cases** |
|  | **per 100,000 persons** | **%** |  | **per 100,000 persons** | **%** |  |
| Crohn's disease | 3.7 | 0.004% | 43,525 | 3.4 | 0.003% | 39,527 |
| Ulcerative colitis | 16.1 | 0.016% | 188,107 | 12.6 | 0.013% | 146,388 |
| Multiple sclerosis | 4.1 | 0.004% | 47,611 | 2.4 | 0.002% | 28,479 |
| Type 1 diabetes | 47.9 | 0.048% | 559,328 | 47.9 | 0.048% | 556,992 |
| Systemic lupus erythematosus | 93.4 | 0.093% | 1,091,075 | 60.3 | 0.060% | 701,170 |
| Rheumatoid arthritis | 104.4 | 0.104% | 1,219,173 | 354.0 | 0.354% | 4,116,090 |
| Graves' disease | 450.3 | 0.450% | 5,257,823 | 538.3 | 0.538% | 6,259,459 |
| Autoimmune thyroiditis | 2321.5 | 2.322% | 27,108,475 | 1692.0 | 1.692% | 19,675,340 |
| Total | **3041.5** | **3.041%** | **35,515,117** | **2710.9** | **2.711%** | **31,523,443** |

*In calculating the conservative estimate, the fixed-effects pooled prevalence was used for all conditions except for type 1 diabetes, where only one study estimate was included in the meta-analysis. This study estimate was used to calculate the expected cases. The population was the 2021 Chinese adult population aged 15 and over. † In calculating the liberal estimate, the random-effects pooled prevalence was used where available; for Crohn’s disease and ulcerative colitis only a fixed-effects estimate was available, and this was used instead; for type 1 diabetes only one study estimate was available, so this was used to calculate the expected cases

**Supplementary Figure 1 Funnel plot**


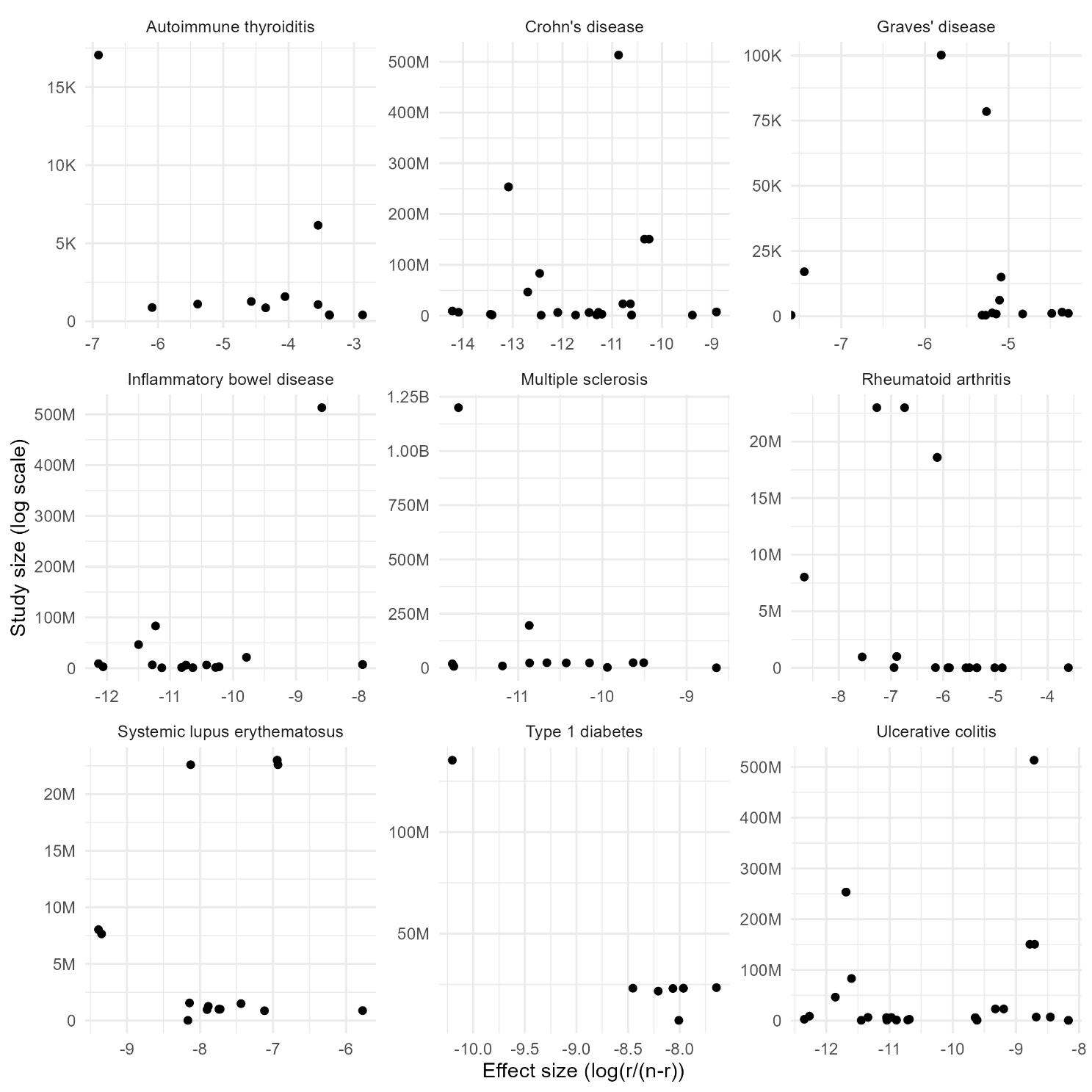


**References (articles identified by systematic review, in alphabetical order, by surname)**

Y.-C. Chen, F.-P. Chen, T.-J. Chen, L.-F. Chou, S.-J. Hwang, Patterns of traditional Chinese medicine use in patients with inflammatory bowel disease: a population study in Taiwan, Hepato-Gastroenterology 55 (82) (2008) 467–470.

Q. Cheng, L. Miao, J. Zhang, et al., A population-based survey of multiple sclerosis in Shanghai, China, Neurology 68 (18) (2007) 1495–1500.

Y.-M. Chiu, C.-H. Lai, Nationwide population-based epidemiologic study of systemic lupus erythematosus in Taiwan, Lupus 19 (10) (2010) 1250–1255.

D.K.L. Chow, R.W.L. Leong, K.K.F. Tsoi, et al., Long-term follow-up of ulcerative colitis in the Chinese population, Am J Gastroenterol 104 (3) (2009) 647–654.

C.-H. Chuang, S.-H. Lin, C.-Y. Chen, B.-S. Sheu, A.-W. Kao, J.-D. Wang, Increasing incidence and lifetime risk of inflammatory bowel disease in Taiwan: a nationwide study in a low-endemic area 1998-2010, Inflamm Bowel Dis 19 (13) (2013) 2815–2819.

S.-M. Dai, X.-H. Han, D.-B. Zhao, Y.-Q. Shi, Y. Liu, J.-M. Meng, Prevalence of rheumatic symptoms, rheumatoid arthritis, ankylosing spondylitis, and gout in Shanghai, China: a COPCORD study, J Rheumatol 30 (10) (2003) 2245–2251.

F. Gu, G. Ding, X. Lou, et al., Incidence of thyroid diseases in Zhejiang Province, China, after 15 years of salt iodization, J Trace Elem Med Biol 36 (2016) 57–64.

C.Y. Hsu, L.S. Ro, L.J. Chen, et al., Epidemiology, treatment patterns and healthcare utilizations in multiple sclerosis in Taiwan, Sci Rep 11 (1) (2021) 7727.

L. Jiang, B. Xia, J. Li, et al., Retrospective survey of 452 patients with inflammatory bowel disease in Wuhan city, Central China, Inflamm Bowel Dis 12 (3) (2006) 212–217.

Y.-D. Jiang, C.-H. Chang, T.-Y. Tai, J.-F. Chen, L.-M. Chuang, Incidence and prevalence rates of diabetes mellitus in Taiwan: analysis of the Nationwide health insurance database, J Formosan Med Assoc 111 (11) (2012) 599–604.

C.-J. Kuo, K.-H. Yu, L.-C. See, et al., The trend of inflammatory bowel diseases in Taiwan: a population-based study, Dig Dis Sci 60 (8) (2015) 2454–2462.

C.-F. Kuo, S.-F. Luo, L.-C. See, I.-J. Chou, H.-C. Chang, K.-H. Yu, Rheumatoid arthritis prevalence, incidence, and mortality rates: a nationwide population study in Taiwan, Rheumatol Int 33 (2) (2013) 355–360.

X.F. Lai, Z.K. Liu, P. Shen, et al., Epidemiological study of incidence of systematic lupus erythematosus in Yinzhou, Ningbo, 2016-2021, Zhonghua Liu Xing Bing Xue Za Zhi 44 (7) (2023) 1080–1085.

C.H. Lai, M.S. Lai, K.L. Lai, H.H. Chen, Y.M. Chiu, Nationwide population-based epidemiologic study of rheumatoid arthritis in Taiwan, Clin Exp Rheumatol 30 (3) (2012) 358–363.

C.-H. Lai, H.-F. Tseng, Population-based epidemiological study of neurological diseases in Taiwan: I. Creutzfeldt-Jakob disease and multiple sclerosis, Neuroepidemiology 33 (3) (2009) 247–253.

P.C. Langley, R. Mu, M. Wu, P. Dong, B. Tang, The impact of rheumatoid arthritis on the burden of disease in urban China, J Med Econ 14 (6) (2011) 709–719.

K.K. Lau, L.K.S. Wong, L.S.W. Li, Y.W. Chan, H.L. Li, V. Wong, Epidemiological study of multiple sclerosis in Hong Kong Chinese: questionnaire survey, Hong Kong Medical Journal = Xianggang yi xue za zhi 8 (2) (2002) 77–80.

K.K. Lau, W.W.-Y. Wong, B. Sheng, et al., The clinical course of multiple sclerosis patients in Hong Kong, J Neurol Sci 268 (1) (2008) 78–82.

P.Y. Leong, J.Y. Huang, J.Y. Chiou, Y.C. Bai, J.C. Wei, The prevalence and incidence of systemic lupus erythematosus in Taiwan: a nationwide population-based study, Sci Rep 11 (1) (2021) 5631.

R.W.L. Leong, J.Y. Lau, J.J.Y. Sung, The epidemiology and phenotype of Crohn’ s disease in the Chinese population, Inflamm Bowel Dis 10 (5) (2004) 646–651.

R. Li, J. Sun, L.-M. Ren, et al., Epidemiology of eight common rheumatic diseases in China: a large-scale cross-sectional survey in Beijing, Rheumatology 51 (4) (2012) 721–729.

Y. Li, C. Li, H. Yin, A large scale epidemiological survey of Graves’ disease in Daqing area, Chin Med J (Engl) 113 (1) (2000) 31–34.

C.M. Liao, W.Y. Kuo, P.T. Kung, C.H. Chuan, W.C. Tsai, Epidemiological investigation of multiple sclerosis and related medical utilisation in Taiwan, Mult Scler 28 (8) (2022) 1198–1208.

W.-H. Lin, M.-C. Wang, W.-M. Wang, et al., Incidence of and mortality from type I diabetes in Taiwan from 1999 through 2010: a nationwide cohort study, PloS One 9 (1) (2014) e86172.

Y.-C. Lin, S.-J. Liang, Y.-H. Liu, et al., Tuberculosis as a risk factor for systemic lupus erythematosus: results of a nationwide study in Taiwan, Rheumatol Int 32 (6) (2012) 1669–1673.

Z. Liu, Z. Nie, Y. Lu, et al., Prevalence of multiple sclerosis in Guangzhou, China: a population-based case-finding prospective study, Mult Scler Relat Disord 68 (2022) 104151.

C. Liu, Y.C. Yuan, M.N. Guo, et al., Incidence of type 1 diabetes may be underestimated in the Chinese population: evidence from 21.7 million people between 2007 and 2017, Diabetes Care 44 (11) (2021) 2503–2509.

K.-H. Lok, H.-G. Hung, C.-H. Ng, et al., Epidemiology and clinical characteristics of ulcerative colitis in Chinese population: experience from a single center in Hong Kong, J Gastroenterol Hepatol 23 (3) (2008) 406–410.

K.H. Lok, H.G. Hung, C.H. Ng, K.K. Li, K.F. Li, M.L. Szeto, The epidemiology and clinical characteristics of Crohn’s disease in the Hong Kong Chinese population: experiences from a regional hospital, Hong Kong Medical Journal=Xianggang yi xue za zhi 13 (6) (2007) 436–441.

A.O.Y. Luk, C. Ke, E.S.H. Lau, et al., Secular trends in incidence of type 1 and type 2 diabetes in Hong Kong: a retrospective cohort study, PLoS Med 17 (2) (2020) e1003052.

C.C. Mok, To CH, L.Y. Ho, K.L. Yu, Incidence and mortality of systemic lupus erythematosus in a southern Chinese population, 2000-2006, J Rheumatol 35 (10) (2008) 1978–1982.

C.C. Mok, C.S. Lau, Lupus in Hong Kong Chinese, Lupus 12 (9) (2003) 717–722.

S.C. Ng, G.G. Kaplan, W. Tang, et al., Population density and risk of inflammatory bowel disease: a prospective population-based study in 13 countries or regions in Asia-Pacific, Am J Gastroenterol 114 (1) (2019) 107–115.

S.C. Ng, W.K. Leung, H.Y. Shi, et al., Epidemiology of inflammatory bowel disease from 1981 to 2014: results from a territory-wide population-based registry in Hong Kong, Inflamm Bowel Dis 22 (8) (2016) 1954–1960.

L.-C. See, C.-F. Kuo, I.-J. Chou, M.-J. Chiou, K.-H. Yu, Sex- and age-specific incidence of autoimmune rheumatic diseases in the Chinese population: a Taiwan population-based study, Semin Arthritis Rheum 43 (3) (2013) 381–386.

Z. Shan, L. Chen, X. Lian, et al., Iodine status and prevalence of thyroid disorders after introduction of mandatory universal salt iodization for 16 years in China: a cross-sectional study in 10 cities, Thyroid 26 (8) (2016) 1125–1130.

Y.-J. Sheen, C.-C. Hsu, Y.-D. Jiang, C.-N. Huang, J.-S. Liu, W.H.-H. Sheu, Trends in prevalence and incidence of diabetes mellitus from 2005 to 2014 in Taiwan, J Formosan Med Assoc 118 (9214933) (2019) S66–S73.

F. Shi, K. Gu, W. Lu, et al., Study on the prevalence of arthritis and relevant factors in Shanghai, Zhonghua liu xing bing xue za zhi = Zhonghua liuxingbingxue zazhi 24 (12) (2003) 1136–1140.

H.Y. Shi, F.K.L. Chan, W.K. Leung, et al., Natural history of elderly-onset ulcerative colitis: results from a territory-wide inflammatory bowel disease registry, J Crohns Colitis 10 (2) (2016) 176–185.

J. Sun, D.-w. Liu, Z.-s. Liu, W. Li, G.-k. Li, J.-w. Zhang, Epidemiology of rheumatoid arthritis in middle-aged and elderly population in Luohe City, Henan Province, Zhonghua Yi Xue Za Zhi 93 (41) (2013) 3309–3311.

W. Teng, Z. Shan, X. Teng, et al., Effect of iodine intake on thyroid diseases in China, N Engl J Med 354 (26) (2006) 2783–2793.

D.-C. Tian, C. Zhang, M. Yuan, et al., Incidence of multiple sclerosis in China: a nationwide hospital-based study, Lancet Regional Health-Western Pacific 1 (2020) 100010.

C.-P. Tsai, C.-L. Yuan, H.-Y. Yu, C. Chen, Y.-C. Guo, D.-E. Shan, Multiple sclerosis in Taiwan, J Chin Med Assoc JCMA 67 (10) (2004) 500–505.

S. Wan, M. Qu, H. Wu, et al., Autoimmune thyroid diseases after 25 years of universal salt iodisation: an epidemiological study of Chinese adults in areas with different water iodine levels, Br J Nutr 124 (8) (2020) 853–864.

C. Wang, Y. Li, D. Teng, et al., Hyperthyroidism prevalence in China after universal salt iodization, Front Endocrinol (Lausanne) 12 (2021) 651534.

S.-C. Wei, M.-H. Lin, C.-C. Tung, et al., A nationwide population-based study of the inflammatory bowel diseases between 1998 and 2008 in Taiwan, BMC Gastroenterol 13 (100968547) (2013) 166.

S.-C. Wei, M.-J. Shieh, M.-C. Chang, Y.-T. Chang, C.-Y. Wang, J.-M. Wong, Long-term follow-up of ulcerative colitis in Taiwan, J Chin Med Assoc JCMA 75 (4) (2012) 151–155.

J. Weng, Z. Zhou, L. Guo, et al., Incidence of type 1 diabetes in China, 2010-13: population based study, BMJ (Clinical Res Ed) 360 (8900488) (2018) j5295.

Q. Wu, M.P. Rayman, H. Lv, et al., Low population selenium status is associated with increased prevalence of thyroid disease, J Clin Endocrinol Metab 100 (11) (2015) 4037–4047.

L. Xu, B. He, Y. Sun, et al., Incidence of Inflammatory Bowel Disease in Urban China: A Nationwide Population-based Study. Clin Gastroenterol Hepatol. (2023) Dec;21(13):3379-3386.e29.

L. Xu, L. Chen, S. Wang, et al., Urban prevalence of multiple sclerosis in China: a population-based study in six provinces, Eur J Neurol 28 (5) (2021) 1636–1644.

H. Yang, R. Zhou, X. Bai, et al., Trend and geographic variation in incidence and prevalence of inflammatory bowel disease in regions across China: a Nationwide employee study between 2013 and 2016, Front Med (Lausanne) 9 (2022) 900251.

H. Yang, Y. Li, W. Wu, et al., The incidence of inflammatory bowel disease in northern China: a prospective population-based study, PloS One 9 (7) (2014) e101296.

K.-W. Yeh, C.-H. Yu, P.-C. Chan, J.-T. Horng, J.-L. Huang, Burden of systemic lupus erythematosus in Taiwan: a population-based survey, Rheumatol Int 33 (7) (2013) 1805–1811.

K.H. Yu, L.C. See, C.F. Kuo, I.J. Chou, M.J. Chou, Prevalence and incidence in patients with autoimmune rheumatic diseases: a nationwide population-based study in Taiwan, Arthritis Care Res 65 (2) (2013) 244–250.

S.-Y. Zeng, Y. Gong, Y.-P. Zhang, et al., Changes in the prevalence of rheumatic diseases in Shantou, China, in the past three decades: a COPCORD study, PloS One 10 (9) (2015) e0138492.

Z. Zeng, Z. Zhu, Y. Yang, et al., Incidence and clinical characteristics of inflammatory bowel disease in a developed region of Guangdong Province, China: a prospective population-based study, J Gastroenterol Hepatol 28 (7) (2013) 1148–1153.

X.-G. Zeng, B. Chen, F. Zeng, et al., Study on the prevalence rate of rheumatoid arthritis in Zhuang nationality population from Nanning, Guangxi, Zhonghua li xing bing xue za zhi = Zhonghua liuxingbingxue zazhi 28 (11) (2007) 1127–1129.

Q. Zeng, S. Huang, R. Chen, 10-year epidemiological study on rheumatic diseases in Shantou area, Zhonghua Nei Ke Za Zhi 36 (3) (1997) 193–197.

N. Zhang, R. Wigley, Q. Zeng, Rheumatic diseases in China, Zhonghua Nei Ke Za Zhi 34 (2) (1995) 79–83.

J. Zhao, S.C. Ng, Y. Lei, et al., First prospective, population-based inflammatory bowel disease incidence study in mainland of China: the emergence of “western” disease, Inflamm Bowel Dis 19 (9) (2013) 1839–1845.

Y.-F. Zou, C.C. Feng, J.-M. Zhu, et al., Prevalence of systemic lupus erythematosus and risk factors in rural areas of Anhui Province, Rheumatol Int 34 (3) (2014) 347–356.
